# Sudden Cardiac Death Due to Deficiency of the Mitochondrial Inorganic Pyrophosphatase PPA2

**DOI:** 10.1016/j.ajhg.2016.06.027

**Published:** 2016-08-11

**Authors:** Hannah Kennedy, Tobias B. Haack, Verity Hartill, Lavinija Mataković, E. Regula Baumgartner, Howard Potter, Richard Mackay, Charlotte L. Alston, Siobhan O’Sullivan, Robert McFarland, Grainne Connolly, Caroline Gannon, Richard King, Scott Mead, Ian Crozier, Wandy Chan, Chris M. Florkowski, Martin Sage, Thomas Höfken, Bader Alhaddad, Laura S. Kremer, Robert Kopajtich, René G. Feichtinger, Wolfgang Sperl, Richard J. Rodenburg, Jean Claude Minet, Angus Dobbie, Tim M. Strom, Thomas Meitinger, Peter M. George, Colin A. Johnson, Robert W. Taylor, Holger Prokisch, Kit Doudney, Johannes A. Mayr

**Affiliations:** 1 Molecular Pathology Laboratory, Canterbury Health Laboratories, Canterbury District Health Board, Christchurch 8140, New Zealand; 2 Department of Pathology, University of Otago, Christchurch 8140, New Zealand; 3 Institute of Human Genetics, Helmholtz Zentrum München – German Research Center for Environmental Health, 85764 Neuherberg, Germany; 4 Institute of Human Genetics, Technische Universität München, 81675 Munich, Germany; 5 Section of Ophthalmology & Neurosciences, Leeds Institute of Biomedical and Clinical Sciences, University of Leeds, Leeds LS9 7TF, UK; 6 Department of Pediatrics, Paracelsus Medical University Salzburg, 5020 Salzburg, Austria; 7 Metabolic Unit, University Children’s Hospital Basel (UKBB), 4056 Basel, Switzerland; 8 Wellcome Trust Centre for Mitochondrial Research, Institute of Neuroscience, Newcastle University, Newcastle upon Tyne NE2 4HH, UK; 9 Department of Metabolic Paediatrics, Royal Hospital for Sick Children, Belfast BT12 6BA, UK; 10 Department of Clinical Biochemistry, Royal Victoria Hospital, Belfast BT12 6BA, UK; 11 Department of Pathology, Royal Victoria Hospital, Belfast BT12 6BA, UK; 12 Department of Cardiology, Christchurch Hospital, Canterbury District Health Board, Christchurch 8140, New Zealand; 13 University of Queensland School of Medicine, Brisbane, QLD 4006, Australia; 14 Department of Anatomical Pathology, Christchurch Hospital, Canterbury District Health Board, Christchurch 8140, New Zealand; 15 Department of Life Sciences, Brunel University London, Uxbridge, Middlesex UB8 3PH, UK; 16 Department of Pediatrics, Nijmegen Center for Mitochondrial Disorders, Radboud University Medical Centre, 6500HB Nijmegen, the Netherlands; 17 Department of Neonatology UKBB Bruderholz, University Children’s Hospital Basel, 4056 Basel, Switzerland; 18 Yorkshire Regional Genetics Service, Chapel Allerton Hospital, Leeds LS7 4SA, UK; 19 DZHK (German Centre for Cardiovascular Research), partner site Munich Heart Alliance, 80802 Munich, Germany

## Abstract

We have used whole-exome sequencing in ten individuals from four unrelated pedigrees to identify biallelic missense mutations in the nuclear-encoded mitochondrial inorganic pyrophosphatase (*PPA2*) that are associated with mitochondrial disease. These individuals show a range of severity, indicating that *PPA2* mutations may cause a spectrum of mitochondrial disease phenotypes. Severe symptoms include seizures, lactic acidosis, cardiac arrhythmia, and death within days of birth. In the index family, presentation was milder and manifested as cardiac fibrosis and an exquisite sensitivity to alcohol, leading to sudden arrhythmic cardiac death in the second decade of life. Comparison of normal and mutant PPA2-containing mitochondria from fibroblasts showed that the activity of inorganic pyrophosphatase was significantly reduced in affected individuals. Recombinant PPA2 enzymes modeling hypomorphic missense mutations had decreased activity that correlated with disease severity. These findings confirm the pathogenicity of *PPA2* mutations and suggest that PPA2 is a cardiomyopathy-associated protein, which has a greater physiological importance in mitochondrial function than previously recognized.

## Main Text

Inorganic pyrophosphate (PPi, also termed diphosphate) is formed by several important nucleotide triphosphate-dependent reactions necessary for DNA, RNA, protein, and lipid synthesis. Pyrophosphate has to be hydrolyzed to orthophosphate (Pi). An enzyme that catalyzes this reaction is termed inorganic pyrophosphatase (PPA [Enzyme Commission number EC 3.6.1.1]) and provides Pi for biomolecules via synthesis of ATP, the terminal product of cellular energy metabolism. PPAs are found in all kingdoms of life. Type I enzymes present in *Escherichia coli* and eukaryotes depend on divalent ions, preferably Mg^2+^ ions.^1^ Humans, similar to the yeast *Saccharomyces cerevisiae*, have two PPAs that share sequence homology. These comprise a cytoplasmic soluble PPA1 and a mitochondrial-located PPA2. For the latter it has been proposed that the soluble catalytic part binds to a yet uncharacterized inner mitochondrial membrane protein.^2^ Knockout of the cytoplasmic *PPA1* (MIM: 179030) ortholog *IPP1* leads to a loss of viability in yeast.^3^, ^4^ Knockout of the mitochondrial *PPA2* results in a growth defect on non-fermentable carbon sources and loss of mitochondrial DNA in *S. cerevisiae*.^3^

We have identified hypomorphic missense mutations in the human gene of the mitochondrial inorganic pyrophosphatase encoded by *PPA2* (MIM: 609988) in a multicenter study by exploring undiagnosed cases with presumed mitochondrial disease using whole-exome sequencing (WES). In agreement with the Declaration of Helsinki, informed consent for genetic and biochemical studies was obtained from all study participants or their guardians. The study was approved by the Ethics Committee of the Technische Universität München and South Yorkshire Research Ethics Committee.

**Figure 1:**
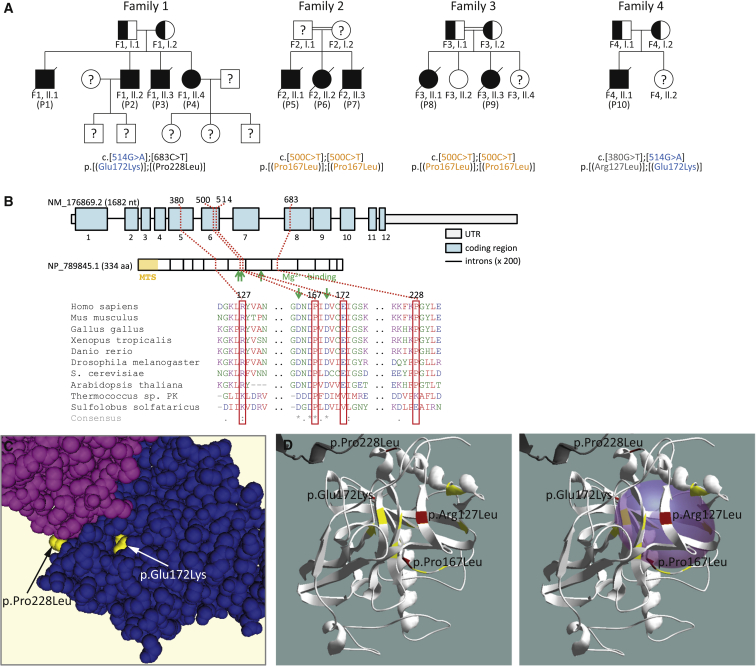
Pedigree Structure, *PPA2* Genomic Organization Conservation, and Family 1 Variant Modeling (A) Pedigrees of four families identified with mutations in *PPA2* (GenBank: NM_176869.2) encoding the mitochondrial inorganic pyrophosphatase. Individuals with a question mark have not been tested. Mutations found in more than one family are colored. (B) Location of mutations within the gene, and phylogenetic conservation of the predicted missense mutations. (C) Space fill model showing position of p.Pro228 at boundary of dimers and p.Glu172 in the active site produced in CN3D with reference PDB: 1M38. (D) Left: Structural model of one molecule of PPA2 showing the position of four mutations in folded structure (red). Residues that are known to be critical to PPA2 function in *S. cerevisiae* are highlighted in yellow.^19^ Right: Space fill of the PPA2 active site showing three substitutions are located at the surface of the active site. Models produced using Swiss-PdbViewer^20^ (with reference PDB: 1M38).

**Figure 2:**
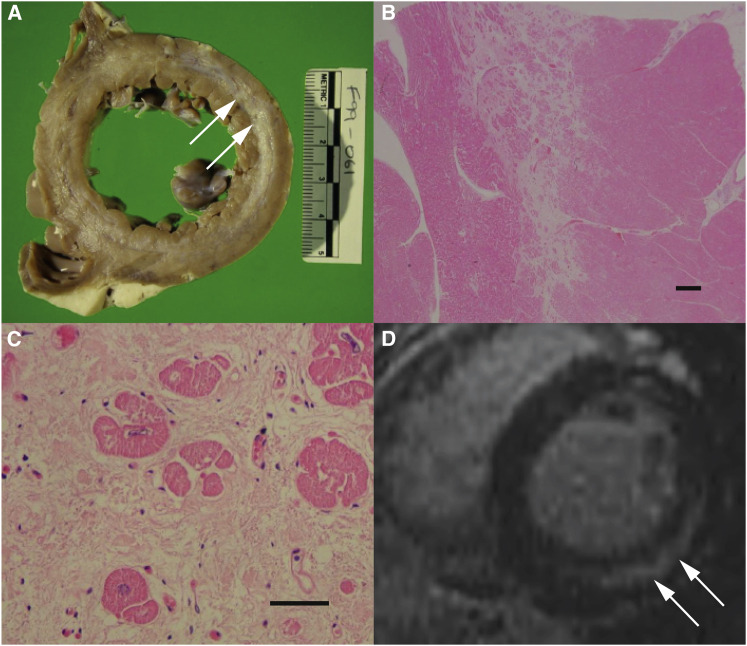
Cardiac Fibrosis in PPA2 Deficiency (A) Affected individual P3, post mortem section through left ventricle showing a virtually circumferential lamina of scarring in midmyocardium with focal subendocardial involvement. Fibrosis is marked by arrows. (B and C) Low-power (B) (bar equals 1 mm) and high-power (C) (bar equals 25 μm) microscopy of the posterior freewall of the left ventricle showing prominent midmyocardial loose fibrosis in P3 (hematoxylin and eosin staining). (D) Cardiac MRI showing prominent midmyocardial fibrosis in affected individual P4 (at 25 years of age), marked by arrows.

Family 1 consists of four affected individuals (P1–P4) born to healthy unrelated parents of European descent from New Zealand (Figure 1). This family was identified after the sudden death of two children. The first child, P1, was well until the age of 15 years when he collapsed and died after ingestion of a small volume of beer. He had no prior cardiac symptoms, but had exhibited exquisite sensitivity to alcohol in medicine and food, which was common to all four siblings in childhood. This was manifest by pallor and severe chest and arm pain after consumption of small amounts of alcohol (<0.1 g ethanol). The only abnormalities observed post mortem were in the heart, with both ventricles found to be slightly dilated. A diagnosis of myocarditis and sudden arrhythmic cardiac death was made. Individual P3 died suddenly at 20 years of age after ingestion of a small amount of alcohol (approx. 10 g ethanol). He was previously well and had no prior cardiac symptoms, but had also been exquisitely sensitive to alcohol. At post mortem examination the heart weighed 395 g (normal 300 g). The left ventricle was dilated with a virtually circumferential lamina of scarring in mid-myocardium. Microscopic examination revealed very widespread, mostly mature scarring of mid-myocardium in all sectors (Figure 2). Two living siblings, P2 and P4 (currently 38 and 34 years of age, respectively) were assessed based on their family history and their sensitivity to alcohol. Cardiac MRI showed marked mid-myocardial fibrosis in both siblings (P4 shown in Figure 2). They subsequently each received an implantable defibrillator for primary prophylaxis of sudden arrhythmic cardiac death, although no events have occurred to date. Despite extensive investigations into the cause of sudden death in this family over a period of >20 years, no definitive diagnosis was made (for more clinical details see Supplemental Data).

Whole-exome sequencing (see Table S1 for details) was performed on the two living siblings to elucidate the underlying molecular defect. Given that both parents appeared unaffected, we searched for rare non-synonymous variants common to the two affected children in a recessive disease model of inheritance. Four candidate genes were identified with compounding missense mutations and with an association to cardiomyopathy and/or mitochondrial function: *KCNJ12* (MIM: 602323), *TTN* (MIM: 188840), *AARS2* (MIM: 612035), and *PPA2*. Of these four genes, variants in all but *PPA2* were excluded based on non-segregation with disease (Table S2). Both affected children were compound heterozygous for *PPA2* mutations c.[514G>A];[683C>T] causing the predicted coding changes p.[Glu172Lys];[Pro228Leu], with each parent carrying one mutation. Sanger sequencing confirmed compound heterozygosity of *PPA2* mutations in the two deceased individuals, establishing the same genotype for all four affected individuals (Figure S1).

We considered that PPA2 dysfunction was the likely underlying cause of sudden cardiac death in our index family. We identified an additional three families with a further six affected individuals harboring compound heterozygous or homozygous *PPA2* mutations (Figure 1) in large exome datasets from individuals suspected of a disorder in mitochondrial energy metabolism. Family 2 (c.[500C>T];[500C>T], p.[Pro167Leu];[Pro167Leu]) (GenBank: NM_176869.2) comprises three affected siblings born to consanguineous parents from Sri Lanka. Family 3 (c.[500C>T];[500C>T], p.[Pro167Leu];[Pro167Leu]) consists of two affected and two healthy siblings born to consanguineous parents of Pakistani origin. Family 4 (c.[380G>T];[514G>A], p.[Arg127Leu];[Glu172Lys]) has one affected and one healthy sibling born to unrelated healthy parents from the United Kingdom.

Unlike the individuals from family 1, all the affected individuals in these three families presented with classical mitochondrial disease symptoms and died within the first 2 years of life of cardiac failure (Table 1). The identification of these individuals suggests that a spectrum of severity is conferred by different biallelic *PPA2* mutations. In affected individuals homozygous for c.500C>T (p.Pro167Leu), the clinical presentation involved lactic acidosis, seizures, hypotonia, and cardiac arrhythmia within the first months of life. Myocyte loss, disarray, or fibrosis was present in all individuals. Respiratory chain function varied from normal to moderate reduction in complex I and IV activities in cardiac tissue and was normal in fibroblasts and skeletal muscle tissue. The individual harboring compound heterozygous c.[380G>T];[514G>A], p.[Arg127Leu];[Glu172Lys] mutations first presented with short seizures at 10 months and developed dilated cardiomyopathy and multiorgan failure at 1 year, necessitating intensive care for several weeks. All affected individuals died from cardiac failure after sudden deterioration. Interestingly, both individuals from family 3 and the affected individual from family 4 had viral infections at the time of hospital admission before their final heart failure (Figure S2). In the older siblings from family 2, vomiting (among other symptoms of metabolic decompensation prior to admission) was reported, one of them having loose stools once, but viral infection was not confirmed (Table 1). Clinical case histories of all affected individuals are provided in the Supplemental Data.

Western blotting showed normal amounts of PPA2 protein in fibroblast mitochondria from individuals P5, P6, and P7 but decreased amount in P9 (Figure S3). In autopsy muscle of P9, the amount of PPA2 protein was decreased, although it appeared to be normal in P6, who carried the same *PPA2* mutation (Figure S4). In heart autopsy material from P10, we noted decreased PPA2 levels as well as decreased levels of a complex I structural protein (subunit NDUFS4), correlating with the observed decrease in complex I activity in this tissue (Figure S5). In the cardiac autopsy sample of P7, PPA2 and complex I subunit levels were decreased as was the expression of the mitochondrial marker proteins porin and citrate synthase, suggestive of a more general reduction of mitochondrial number possibly due to changes in tissue composition (Figure S5).

All four missense variants involve residues of high evolutionary conservation (Figure 1) and are predicted to have a pathogenic effect on PPA2 function in silico (SIFT, PolyPhen-2, and MutationTaster) (Table S3). The high homology between the human and yeast (*S. cerevisiae*) PPA proteins facilitated predictive modeling of these human variants based on the known yeast structure of the cytosolic/nuclear pyrophosphatase IPP1 (MMDB ID: 21720; PDB: 1M38) (Figure 1). Glutamine to lysine substitution at residue 172 is predicted to disrupt at least three hydrogen bonds between interacting protein chains near the surface of the enzyme’s active site. Any disruption of the active site may impair enzymatic function of PPA2. A substitution of proline to leucine at residue 228, located on the outside surface where dimer association occurs, is also predicted to disrupt the secondary structure of PPA2. Proline is a known peptide turning point amino acid and is likely to affect the orientation of the two helices between which it lies in this enzyme. We suggest that disruption of the conformation of the outer surface may impair correct dimerization of PPA2 molecules.

All four *PPA2* mutations identified in our cohort are present in the Exome Aggregation Consortium (ExAC) database (12/2015) at a frequency < 0.005, equating to 59/60,400 individuals heterozygous for p.Glu172Lys, 30/60,134 individuals heterozygous for p.Pro228Leu, 20/60,677 individuals heterozygous for p.Arg127Leu, and 3/60,457 individuals heterozygous for p.Pro167Leu (Table S1). None of these *PPA2* variants is reported in a homozygous state in ExAC, the NHLBI Exome Sequencing Project (ESP6500) database, or 7,000 control exomes of an in-house database (Munich). Due to the complete growth defect of yeast PPA2 knockouts on non-fermentable carbon sources, it can be speculated that biallelic loss-of-function mutations of *PPA2* are incompatible with life in humans. In total, 13 LOF variants (found in 18 alleles) are published in the ExAC database and furthermore ExAC contains 71 different missense mutations (in 237 alleles) with a SIFT score ≤ 0.05 (cut-off for mutations to be considered pathogenically relevant). The cumulative heterozygous carrier frequency of these likely pathogenic *PPA2* mutations is 0.0024, which would result in a calculated prevalence for compound heterozygous or homozygous pathogenic *PPA2* mutations of 0.58 per 100,000 (1 in 170,000).

In order to investigate effects of PPA2 deficiency on the cellular metabolism, we measured oxygen consumption rate (OCR) by micro scale respirometry (XF96 Seahorse Biosciences).^5^ Basal respiration and oligomycin-inhibited OCR was similar in affected individuals (P5, P6, and P7) but after the addition of the mitochondrial uncoupler FCCP, a higher activity was observed in affected individuals compared to control subjects. The difference between basal and FCCP-stimulated OCR, termed reserve respiratory capacity (RRC), was twice as high in PPA2-deficient fibroblasts compared to controls (Figures S6A and S6B). High RRC observed in PPA2-deficient cells might be due to a limitation in ATP synthesis because of insufficient Pi supply within mitochondria (Figure S7). Actually, the investigation of cells with proven ATP synthase deficiency due to mutations in either *TMEM70*^6^ (MIM: 612418) or *ATP5E*^7^ (MIM: 606153) revealed a similar OCR-profile with high RRC. Because high RRC is not a specific finding, further investigations were required to demonstrate decreased ATP synthesis in PPA2 deficiency.

**Figure 3:**
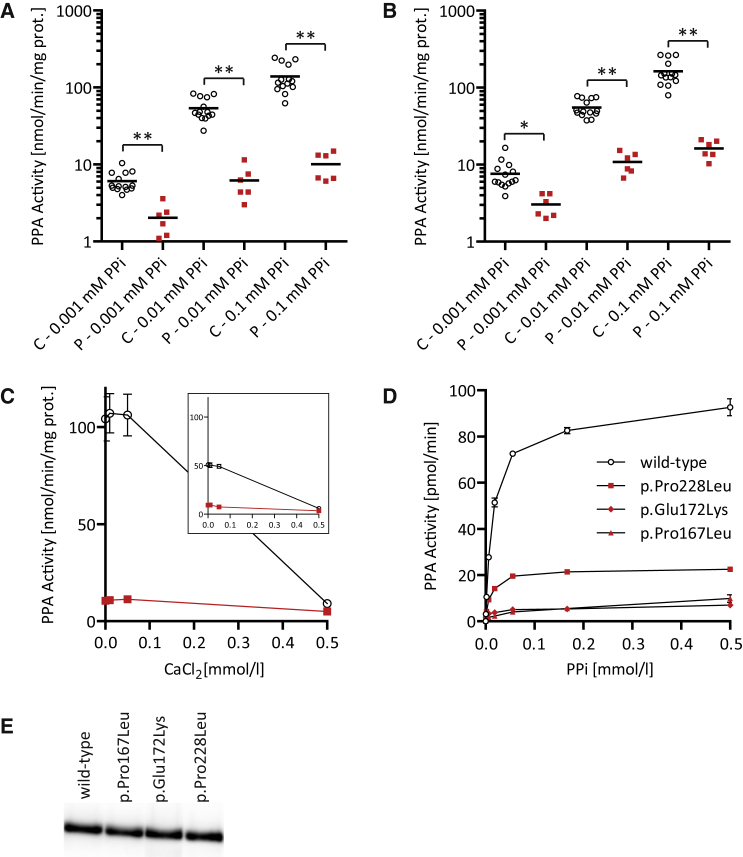
Inorganic Pyrophosphatase Activity in Fibroblast Mitochondria and Recombinant Enzymes (A and B) Activity of inorganic pyrophosphatase in different fibroblast mitochondria isolations from affected individuals (P) P5, P7, and P9 compared to 14 control subjects (C) at different PPi concentrations and either (A) 0.5 mmol/L MgCl_2_ or (B) 3.0 mmol/L MgCl_2_. (C) Inhibition of inorganic pyrophosphatase in fibroblast mitochondria from affected individual P5 (red squares) and three control subjects (black circles) incubated at 0.5 mmol/L MgCl_2_ and different CaCl_2_ concentrations and either 0.1 mmol/L PPi or 0.01 mmol/L PPi (small insert). (D) Pyrophosphatase activity of equal amounts of recombinant proteins at different PPi concentrations. (E) Protein amount of recombinant PPA2 protein was adjusted by western blot analysis and silver staining (Figure S9). ^∗^p < 0.01, ^∗∗^p < 0.0001 in Student’s unpaired t test. The error bars in this graph indicate the standard error of the mean.

We next determined pyrophosphatase activity in isolated mitochondria from fibroblasts of control and affected individuals, which were prepared from 540 ccm of confluent primary fibroblasts. After harvesting by trypsinization and washing twice with phosphate-buffered saline cells, the weight of the cell pellet was determined. Cells were suspended in the 10-fold amount (e.g., 500 μL per 50 mg of cell pellet) of ice-cold, hypotonic homogenization buffer (10 mmol/L Tris [pH 7.4]) and homogenized by the use of a tight fitting Potter Elvehjem homogenizer. Immediately after homogenization, 1.5 mmol/L sucrose (20% of the homogenization volume) was added to preserve mitochondria. After centrifugation at 600 × *g*, the mitochondria containing supernatant was centrifuged at 10,000 × *g* and the mitochondria-containing pellet was washed twice with SEKT buffer (250 mmol/L sucrose, 2 mmol/L EGTA, 40 mmol/L KCl, 20 mmol/L KCl [pH 7.4]). The mitochondrial pellet was finally suspended in the equal amount of SEKT buffer (50 μL per 50 mg cell pellet) and stored at −80°C prior to further investigations.^7^, ^8^ The hydrolysis of PPi and quantification of orthophosphate (Pi) formed was determined according to previously published methods^9^, ^10^ with minor modifications. The incubation buffer contained 50 mmol/L Tris ([pH 8.0] 0.1 mmol/L EGTA) and the indicated concentrations of MgCl_2_ and PPi. The reaction was started by the addition of enzyme in a final volume of 100 μL, incubated at 37°C, and stopped by the addition of 100 μL reagent A (0.70% (w/v) ammonium heptamolybdate tetrahydrate, 1.26 mol/L H_2_SO_4_), developed by the addition of 40 μL reagent B (0.35% (w/v) polyvinylalcohol, 0.035% (w/v) malachite green oxalate at room temperature for 20 min. The activity of PPA was significantly decreased in isolated fibroblast mitochondria from affected individuals P5, P7, and P9 at each PPi (0.001–0.1 μmol/L) and MgCl_2_ (0.5 or 3.0 mmol/L) concentration investigated (Figures 3A and 3B). Inactivation by CaCl_2_ was similar in affected individuals compared to control subjects (Figure 3C). Fibroblasts from affected individuals P6 and P10 did not grow sufficiently to collect enough cells for the isolation of mitochondria and from individuals P1–P4 and P8 no fibroblasts were available.

For the expression of recombinant human PPA2, wild-type *PPA2* cDNA was cloned into the expression vector pRSET B (Invitrogen) using the cloning sites BamHI and BglII.^11^ The first 96 nucleotides corresponding to the cleavable N-terminal mitochondrial targeting sequence were omitted from the construct. The c.500C>T (p.Pro167Leu), c.514G>A (p.Glu172Lys), and c.683C>T (p.Pro228Leu) variants were introduced into the wild-type *PPA2* sequence by site-directed mutagenesis using Gibson assembly (New England Biolabs) with appropriate primers for PCR amplification (Phusion, New England Biolabs) and the correct coding regions of all four constructs was confirmed by Sanger sequencing. Recombinant protein was expressed in the *Escherichia coli* strain BL21(DE3)pLysS at 37°C starting at OD_600_ of 0.2 and using 1 mmol/L IPTG for 2 hr. The bacterial suspension was harvested and sonified in homogenization buffer and the supernatant was bound to HisPur cobalt spin columns (Thermo).^11^ The amount of the recombinant proteins was determined by western blotting with a human PPA2 antibody (Abcam cat# ab177935). Equal amounts of either wild-type or mutant recombinant PPA2 proteins were used for the pyrophosphatase activity assay. Compared to wild-type, the p.Pro167Leu and p.Glu172Lys variants showed 5%–10% residual activity at PPi substrate concentrations 18–500 μmol/L. The p.Pro228Leu variant had a residual activity of 24%–28% in this concentration range compared to wild-type (Figure 3D). The activities of wild-type and mutants were similarly sensitive to inhibition by Ca^2+^ (data not shown).

As previously reported, *PPA2* knockout strain from *S. cereviseae* is unable to grow on aerobic media.^3^ We also detected a growth defect of *PPA2* knockout yeast on diamide-containing media, which lowers antioxidant concentrations (Figure S8).^12^ These antioxidants protect the cell against reactive oxygen species that are also natural by-products of mitochondrial respiratory chain function. The increased diamide sensitivity of PPA2-deficient yeast therefore suggests reduced levels of antioxidants.

In the case of family 1, our data suggest that p.Pro228Leu is a relatively mild variant, given that PPA2 function is only moderately reduced. This hypothesis is supported by investigation of the activity of recombinant PPA2 enzyme activity. The p.Pro228Leu substitution resulted in a reduction of PPA activity to approximately 25% of wild-type (Figure 3). These individuals show chronic accumulation of cardiac fibrosis, and death occurred after ingestion of alcohol to which they were already known to have acute sensitivity. We propose that alcohol acted as a trigger in these case subjects, whose PPA2 dysfunction created chronic mitochondrial sensitivity and whose hearts were consistently deprived of adequate ATP resulting in fibrosis. Ingestion of alcohol appears to have increased the stress on the already sensitive mitochondria/fibrotic heart, causing cardiac arrhythmia and death. There is a link between alcohol metabolism and inorganic pyrophosphatase function that might underlie the pathology of affected individuals. Ethanol is oxidized to acetaldehyde and further to acetic acid.^13^ Resulting acetic acid has to be activated to acetyl-coenzyme A, which is accompanied by the formation of equimolar amounts of PPi (Figure S7). This esterification reaction is catalyzed by short-chain acyl-CoA synthetases encoded, for example, by *ACSS1* (MIM: 614355), an enzyme with high expression in heart mitochondria.^14^ In cases of severe PPA2 dysfunction, ATP depletion has an acute effect and lactic acidosis and cardiomyopathy occurs prior to chronic damage developing, which could lead to acute symptoms in the presence of secondary triggers. It is interesting to note, however, that both affected individuals in family 3 had a history of vomiting, diarrhea, and seizures prior to admission to hospital, and viral infection (rotavirus [1^st^] and norovirus [2^nd^]) was confirmed in stool samples taken at time of admission. A norovirus infection was also found in P10 from the United Kingdom. This may indicate that a viral stressor was responsible for adversely affecting mitochondrial metabolism in families 3 and 4, in the same way that alcohol was a trigger for arrhythmia in the index family. In the oldest sibling of family 2, there was also some vomiting prior to hospital admission but viral illness was not confirmed. In the two younger siblings, vomiting occurred among other initial symptoms of metabolic compensation, in the youngest sibling who was hospitalized from birth already on the third day of life. Of note, symptoms like vomiting,^15^ diarrhea,^16^ and seizures^17^ are also typical for other disorders of the mitochondrial energy metabolism.

All affected individuals died from cardiac failure. Sudden, unexpected cardiac death was especially observed in P1, P3, P5, P6, and P8. As clearly seen in cardiac MRIs from the two living individuals from family 1, midmyocardial fibrosis is a pre-existing condition (Figure 2) even though no cardiac symptoms were experienced by these individuals. Using late gadolinium enhancement (LGE), myocardial fibrosis can be clearly determined and is also a common finding in other disorders of the mitochondrial energy metabolism such as MELAS (MIM: 540000) due to the common m.3243A>G mutation of the mitochondrial DNA.^18^

In conclusion, we have identified biallelic missense mutations in *PPA2* as cause of mitochondrial cardiomyopathy and sudden cardiac death. This finding highlights a critical role of PPA2 in mitochondrial function and warrants further functional investigation. Importantly, mild mutations in *PPA2* may not have an immediate life-threatening effect until triggered by a stressor such as viral illness or alcohol metabolism, predisposing otherwise healthy individuals to sudden cardiac death. Considering the relatively high frequency of *PPA2* mutations present in the ExAC database, it is important that clinically suspicious individuals are screened for *PPA2* mutations in addition to evidence of heart fibrosis by cardiac MRI. Moreover, application of an implantable cardioverter defibrillator may prevent sudden cardiac death in at-risk individuals who harbor biallelic *PPA2* mutations.

## Figures and Tables

**Table 1:** Genetic and Clinical Findings in Individuals with *PPA2* Variants

**ID**	**Sex (M/F)**	***PPA2* Variants: cDNA (NM_176869.2), Protein (NP_789845.1)**	**OXPHOS Activities (Normal Ranges in Brackets)**	**Clinical Features**			**Other Findings**
				**AO**	**Age at Death**	**Cardiac Phenotype**	
F1, II:1, P1	M	c.[514G>A];[683C>T], p.[Glu172Lys];[Pro228Leu]	ND	4 years	15 years	autopsy: slight dilation of both ventricles; small pale area in the epicardium of the left ventricle, evidence of focal inflammation with neutrophils, lymphocytes, and eosinophils	sensitive to small amounts of alcohol
F1, II:2, P2	M	c.[514G>A];[683C>T], p.[Glu172Lys];[Pro228Leu]	ND	14 years	alive at 38 years	cardiac MRI: myocardial fibrosis; received implantable defibrillator	sensitive to small amounts of alcohol
F1, II:3, P3	M	c.[514G>A];[683C>T], p.[Glu172Lys];[Pro228Leu]	ND	10 years	20 years	autopsy: dilation of the left ventricle, circumferential lamina of scarring in midmyocardium with focal subendocardial involvement; very widespread mostly mature scarring of midmyocardium in all sectors	sensitive to small amounts of alcohol
F1, II:4, P4	F	c.[514G>A];[683C>T], p.[Glu172Lys];[Pro228Leu]	normal in skeletal muscle	9 years	alive at 33 years	cardiac MRI: myocardial fibrosis; received implantable defibrillator.	sensitive to small amounts of alcohol; immunohistochemical studies of skeletal muscle showed changes suggestive of a mild chronic myopathy
F2, II:, P5	M	c.[500C>T];[500C>T], p.[Pro167Leu];[Pro167Leu]	ND	10 days	11 days	autopsy: herds of fresh necrosis mainly of the right heart and interstitial lymphocyte infiltration; electron microscopy: myocard showed mitochondria with degeneration of cristae	elevated plasma lactate levels; tachypnoea and tachycardia; tonic-clonic seizures; death after severe bradycardia
F2, II:2, P6	F	c.[500C>T];[500C>T], p.[Pro167Leu];[Pro167Leu]	normal in skeletal muscle and fibroblasts	14 days	14 days	autopsy: acute and subacute necrosis more pronounced in the right heart more severe than in the left heart; electron microscopy: myocardium showed mitochondria with degeneration of cristae like in P5	metabolic acidosis with elevated plasma lactate levels; tachypneoa; vomiting, generalized seizure; cardio-respiratory decompensation; death 6.5 hr after onset of symptoms; multiple subacute necroses in the semioval center of both cerebral hemispheres
F2, II:3, P7	M	c.[500C>T];[500C>T], p.[Pro167Leu];[Pro167Leu]	normal in skeletal muscle and fibroblasts; heart muscle: LV: CI 4.1 (5.5–51.5) and CIV 64 (73.2–516.6) decreased, RV: CI not detectable, CII 9.0 (25.8–40.7), CIV 42 (73.2-516.6)	3 days	32 days	cardiac tachyarrhythmia; ECG showed hypodynamic right ventricle; autopsy: myocardium without necrosis and inflammatory infiltrations; myocytes with reduced amount of myofibrils; region of fibrosis, partially fat tissue in the right heart	elevated plasma lactate, transaminases, lactate dehydrogenase, creatine kinase (CK), CK-MB, and troponin levels
F3, II:1, P8	F	c.[500C>T];[500C>T], p.[Pro167Leu];[Pro167Leu]	ND	5.5 months	5.5 months	autopsy: evidence for long-standing myocyte loss, increased interstitial myocyte loss, increased interstitial collagen, focal myocyte fiber disarray in the left ventricle and interventricular septum	24 hr history of vomiting and diarrhea, 1× seizures; multiple cardiac arrests; hypoxic injury of the brain; the liver showed mild fatty change
F3, II:3, P9	F	c.[500C>T];[500C>T], p.[Pro167Leu];[Pro167Leu]	normal in skeletal muscle	8 months	11 months	autopsy: extensive fibrosis of the heart muscle	plasma lactate elevated, diarrhea, vomiting; focal seizure then generalized seizure; cardiac arrest
F4, II:1, P10	M	c.[380G>T];[514G>A], p.[Arg127Leu];[Glu172Lys]	normal in skeletal muscle; CI decreased heart muscle 0.026 (0.125 ± 0.048)	10 months	2 years	echocardiography: ejection fraction of 74%, mild left ventricular hypertrophy; autopsy: extensive transmural fibrosis of the left ventricle, acute myocardial ischemia	seizures, urinary organic acids: increased 3-hydroxybutyrate, acetoacetate, and C14:1, C14, C16:1 acylcarnitine elevation in blood.

Abbreviations are as follows: AO, age at onset; F, female; M, male; ND, not determined.

## Web Resources

Cn3D, http://www.ncbi.nlm.nih.gov/Structure/CN3D/cn3d.shtml
Enzyme Nomenclature (Nomenclature Committee of the International Union of Biochemistry and Molecular Biology [NC-IUBMB]), http://www.chem.qmul.ac.uk/iubmb/enzyme/
ExAC Browser, http://exac.broadinstitute.org/
GenBank, http://www.ncbi.nlm.nih.gov/genbank/
MMDB, http://www.ncbi.nlm.nih.gov/structure
MutationTaster, http://www.mutationtaster.org/
NHLBI Exome Sequencing Project (ESP) Exome Variant Server, http://evs.gs.washington.edu/EVS/
OMIM, http://www.omim.org/
PolyPhen-2, http://genetics.bwh.harvard.edu/pph2/
RCSB Protein Data Bank, http://www.rcsb.org/pdb/home/home.do
SIFT, http://sift.bii.a-star.edu.sg/
Swiss PDB Viewer, http://www.expasy.org/spdbv/

## Supplemental Data

Document S1. Supplemental Case Reports, Figures S1–S9, and Tables S1–S3

Document S2. Article plus Supplemental Data
