# Supplementary material for: Sudden Cardiac Death Due to Deficiency of the Mitochondrial Inorganic Pyrophosphatase PPA2
Source: Am J Hum Genet. 2016 Aug 11;99(3):674–82. doi: 10.1016/j.ajhg.2016.06.027 (PMC5011043; doi:10.1016/j.ajhg.2016.06.027)
Supplement: Document S1. Supplemental Case Reports, Figures S1–S9, and Tables S1–S3 [file mmc1.pdf]

The American Journal of Human Genetics, Volume *99*

Supplemental Data

Sudden Cardiac Death Due to Deficiency of the Mitochondrial Inorganic Pyrophosphatase PPA2

Hannah Kennedy, Tobias B. Haack, Verity Hartill, Lavinija Mataković, E. Regula Baumgartner, Howard Potter, Richard Mackay, Charlotte L. Alston, Siobhan O'Sullivan, Robert McFarland, Grainne Connolly, Caroline Gannon, Richard King, Scott Mead, Ian Crozier, Wandy Chan, Chris M. Florkowski, Martin Sage, Thomas Höfken, Bader Alhaddad, Laura S. Kremer, Robert Kopajtich, René G. Feichtinger, Wolfgang Sperl, Richard J. Rodenburg, Jean Claude Minet, Angus Dobbie, Tim M. Strom, Thomas Meitinger, Peter M. George, Colin A. Johnson, Robert W. Taylor, Holger Prokisch, Kit Doudney, and Johannes A. Mayr

Supplemental Case Reports of Four Families with *PPA2* mutations

Family 1 (P1-P4)

The family are of Caucasian origin, living in New Zealand. In the extended family there are no cases with the features seen in the family. The parents are unrelated, well and exhibit none of the features seen in their children. All 4 children are affected by the condition.

P1 (PPA2: c.[514G>A];[683C>T]; p.[Glu172Lys];[Pro228Leu])

Sibling 1; a male born in 1975 who collapsed and died suddenly in 1991 aged 15 years after drinking a small amount of beer.

He was previously well, and had no prior cardiac symptoms, but like all his siblings had been exquisitely sensitive to alcohol. This was manifest by severe chest and arm pain, and pallor following consumption of small amounts of alcohol (<0.1 g) noticed for the first time at the age of 4 years after ingestion of an alcohol-containing cough medicine. At post mortem examination the only abnormalities observed were in the heart with both ventricles being slightly dilated. A small pale area was observed on the epicardium of the left ventricle. Microscopic examination revealed evidence of focal inflammation with neutrophils, lymphocytes and eosinophils. The coronary arteries were normal. A diagnosis of myocarditis and sudden arrhythmic cardiac death was made.

P2 (PPA2: c.[514G>A];[683C>T]; p.[Glu172Lys];[Pro228Leu])

Sibling 3; a male born in 1977, was well with no cardiac symptoms, but exhibited the family sensitivity to alcohol. At the age of 14 years, a medical alcohol challenge resulted in marked pain. He was assessed because of his family history. Physical examination was normal as was ECG, echocardiogram, exercise test and Holter monitor. However an MRI scan showed marked midmyocardial fibrosis. He subsequently received an implantable defibrillator for primary prophylaxis of sudden arrhythmic cardiac death. No events have occurred to date.

P3 (PPA2: c.[514G>A];[683C>T]; p.[Glu172Lys];[Pro228Leu])

A male was born in 1978 died suddenly in 1999 aged 20 years following drinking 10 g of alcohol (one standard drink). He was previously well, and had no prior cardiac symptoms, but had also been exquisitely sensitive to alcohol noticed for the first time at the age of 10 years after accidental ingestion of a small amount of wine. At post mortem examination the only abnormalities observed were in the heart. The heart weighed 395 g (normal 300 g). The left ventricle was dilated with a virtually circumferential lamina of scarring in the midmyocardium with focal sub-endocardial involvement. Microscopic examination revealed very widespread mostly mature scarring of

midmyocardium in all sectors. No ischaemic changes were observed, nor microscopic evidence to suggest acute hypersensitivity or interstitial acute myocarditis (Figure 2). The coronary arteries were normal.

P4 (PPA2: c.[514G>A];[683C>T]; p.[Glu172Lys)];[Pro228Leu])

A female born in 1982, was well with no cardiac symptoms, but exhibited the family sensitivity to alcohol. At the age of 9 years, a medical alcohol challenge resulted in marked pain. She was assessed because of her family history. Physical examination was normal as was ECG, echocardiogram, exercise test and Holter monitor. However an MRI showed marked midmyocardial fibrosis Figure 2). She subsequently received an implantable defibrillator for primary prophylaxis of sudden arrhythmic cardiac death. No events have occurred to date.

Further investigations were undertaken on the surviving siblings to try and elucidate a shared genetic and metabolic basis for this apparently unique constellation of clinical features. Investigations have focused on possible mitochondrial genetic disorder and abnormalities of muscle structural proteins and in particular, laminopathies.

Histopathology of skeletal muscle (from P4) showed no obvious abnormality, although a muscular dystrophy or metabolic disorder could not be excluded. Stains for fat and glycogen were within normal limits. A panel of enzyme stains (including myophosphorylase, nicotinamide adenine dinucleotide (NADH), cytochrome oxidase (COX), succinate dehydrogenase (SDH), myoadenylate deaminase (MADA), aldolase and phosphofructokinase (PFK) was normal. **Immuno-histochemical studies** of skeletal muscle showed changes suggestive of a mild chronic myopathy. Immunostaining for dystrophin, dysferlin, emerin and laminin, however showed no obvious abnormality.

Mitochondrial gene sequencing was undertaken on whole blood, buccal cells and skeletal muscle biopsy from P4. She was found to be homoplasmic for several known mitochondrial polymorphisms and in addition, she was found to be homoplasmic for a novel sequence variant m.9751T>C in the *MT-CO3* gene which would result in the substitution of the normal phenylalanine residue at amino acid position 182 of the protein for a serine, predicted to be a benign substitution. The findings do not unequivocally exclude a mitochondrial disorder since mutations in nuclear genes associated with mitochondrial disorders have not been excluded.

Lamin gene sequencing was undertaken on all 12 exons of the lamin A/C gene (LMNA) and also all coding regions of the lamin-associated protein 2 gene *TMPO* (LAP2) together with flanking intronic

sequences. No mutations were detected. Both surviving children appear to have inherited the paternal LMNA allele (by SNP analysis). Of the two deceased children, one also has the paternal allele (determined by analysis of DNA extracted from FFPE tissue) and the other may also have this allele, although sequencing was inconclusive. This is relevant if the disorder is postulated to be transmitted from the mother.

Urine organic and amino acids on P4 showed no abnormality. **Blood spot acylcarnitine profiling** also showed no abnormality. **Whole blood carnitine** also on P4 was within normal limits at 23 µmol/L (normal 11-58). **Amino acid profile** was normal. It was cautioned, however that a normal acylcarnitine profile does not unequivocally exclude a fatty acid oxidation defect, particularly if a sample has been taken during a period when the she is well.

Acetaldehyde dehydrogenase ALDH2: c.1510G>A (p.Glu504Lys), the alcohol "flushing" polymorphism common in Asian populations, as a possible trigger with the observed alcohol sensitivity – not detected in father, mother or both surviving siblings.

Family 2 (P5-P7)

The family are Tamil people from Sri Lanka living in Switzerland, the parents are first cousins. All 3 children of this family were affected by the same neonatal lethal condition.

P5 (PPA2: c.[500C>T];[500C>T], p.[Pro167Leu];[Pro167Leu])

The boy was born spontaneously in 1996 after an uneventful pregnancy. Birth weight was 2820 g, length 49 cm, head circumference 33.5 cm and Apgar scores 9/10/10. The first days of life were uneventful, mother and child left the hospital on day 6. The baby was breast-fed without problems. On the 10th day of life he vomited once and loose stools were observed. On the next morning the child vomited once more but otherwise his clinical status was unremarkable. On the same afternoon (11th day of life) the child was readmitted with signs of tonic clonic seizures. The muscular hypotonic boy was somnolent and pale. Heart and lung function seemed normal initially. Lactate was elevated at 10.5 mmol/l (normal 0.4-2.8 mmol/l). In the following hours he showed again tonic-clonic seizures, which could be suspended just for a while by treatment with Diazepam and Phenobarbital. Oxygen saturation was persistently low necessitating intubation, but even under artificial respiration this did not improve and generalised tonic clonic seizures persisted. ECG investigation showed a convex ST segment elevation. Few hours later the child died with severe bradycardia.

Investigation of plasma amino acids showed elevated alanine. Investigation of organic acids was normal. Acute myocarditis was suspected.

Investigation of heart autopsy revealed areas of fresh myocardium necrosis mainly of the right heart and interstitial lymphocyte infiltration. Electron microscopy of the heart showed mitochondria with degeneration of cristae but no evidence of viral infection. Microbiological investigations of all body fluids revealed no abnormalities.

P6 (PPA2: c.[500C>T];[500C>T], p.[Pro167Leu];[Pro167Leu])

The girl was born in 1997 after normal gestation and birth with good postnatal adaptation. At the age of 14 days, 2 hours after an uneventful routine check by a paediatrician, the child suddenly deteriorated. She was admitted to the intensive care unit with marked tachypnoea after having vomited twice and having suffered from two generalized seizures. Muscle tone was slightly hypotonic and a marked metabolic acidosis with a blood pH of 6.9 (normal 7.35-7.45), HCO_3 at 4 mmol/l (21-26 mmol/l), lactate 22 mmol/l and pyruvate 253 mmol/l (normal 84-784) was found. There was slight improvement with intravenous bicarbonate and glucose treatment. Additionally a vitamin cocktail was given but subsequently there was cardio-respiratory decompensation and the girl died 6 hours after admission.

Autopsy revealed bilateral acute and subacute necrosis of the myocardium, which was more prominent in the right heart. Electron microscopy of the heart showed mitochondria with degeneration of cristae as seen in the brother (P5). Skeletal muscle was normal. Furthermore multiple subacute necroses in both cerebral hemispheres were found. Investigation of the respiratory chain enzymes and pyruvate dehydrogenase were normal in skeletal muscle and fibroblasts. Organic acids and amino acids in urine and plasma were normal. Screening of mitochondrial DNA from heart and liver did not reveal any pathological findings.

P7 (PPA2: c.[500C>T];[500C>T], p.[Pro167Leu];[Pro167Leu])

This boy was born in 2000 at term after an uncomplicated pregnancy. Birth weight was 3240 g. He was hospitalized in intensive care from the first minute of life and carefully observed. During the first two days he was well, similar to his siblings. Then he started to show progressive sweating, occasional vomiting and elevation of some metabolic parameters as lactate, transaminases, lactate dehydrogenase, creatine kinase, and creatine kinase-MB levels. Assuming that the siblings might have suffered from a defect in the respiratory chain isolated to the cardiac muscle this boy was treated with a cocktail supplement of vitamins usually given in defects of the respiratory chain (coenzyme Q_{10}, riboflavin, vitamins C, E, carnitine, biotin, beta-carotene). Over the next few days he became exhausted during feeding and developed signs of slight cardiac failure. Selective screening for inborn errors did not reveal any pathological findings. Plasma lactate remained within the normal range.

On day 9, the boy was additionally treated with thiamine hydrochloride i.v., 20 mg/d since some symptoms resembled Beri-Beri and marked improvement of his condition was noted. Thiamine was unfortunately discontinued on day 11 since he seemed to be perfectly well. However, his condition worsened again and heart failure became evident with occasional arrhythmia. Levels of troponin and transaminases increased. Echocardiography showed impaired function of the enlarged right ventricle. On day 15 it was decided to supplement him with thiamine 3 x 100 mg/day orally. Heart function improved, and the troponin and transaminase level normalized. However, on day 17 recurring tachycardia occurred, which responded temporarily to adenosine and then to electroconversion, but it recurred over the following days. A regular sinus rhythm could be obtained after treatment with boli of 5-20 mg i.v. thiamine and the boy clinically improved dramatically. However, in spite of thiamine 30-80 mg i.v. daily and propafenone, severe arrhythmia (Hf approx. 140 bpm), which turned out to be ventricular, became a serious problem while cardiac function remained stable and troponin and transaminase levels were normal. On day 30, ventricular arrhythmia persisted and did not respond to lidocaine and electroconversion. The child was neurologically normal for his age, alert and fine. He died in the early morning of the 32nd day of life from untreatable arrhythmia. A final echocardiography showed a hypodynamic right ventricle, while the left ventricle was still in a sufficient status (SF about 28-30%). A defect of thiamine metabolism/transport was considered, however, later studies with fibroblasts (by Ellis Neufeld, Boston) revealed normal thiamine uptake and conversion to thiamine pyrophosphate.

Autopsy revealed a myocardium without necrosis and inflammatory infiltrations. Myocytes with reduced amount of myofibrils were found. In the myocardium of the right heart there was a herd of fibrosis. Investigation of the respiratory chain in autopsy samples of the heart showed a moderate decrease of complex I 4.1 mU/mg protein (normal 5.5-51.5 mU/mg protein) and complex IV 64 mU/mg protein (normal 73.2-516.6) in the left ventricle. In the right ventricle the activity of complex I was not detectable, complex II, 9.0 mU/mg protein (normal 73.2-516.6), and complex IV, 42 mU/mg protein were reduced. Normal activities were found in skeletal muscle and fibroblasts. Investigation of the mitochondrial DNA did not reveal pathogenic mutations.

Family 3 (P8-P9)

This is a multiply consanguineous family of Pakistani origin, living in the UK. The parents are first cousins.

P8 (PPA2: c.[500C>T];[500C>T], p.[Pro167Leu];[Pro167Leu])

Their first child (II:1) was seemingly well with normal growth and development until the age of 5 ½ months. She was then admitted to hospital following a 24 hour history of vomiting and diarrhoea and

had suffered a seizure at home. She had further seizures on arrival at A&E and a poor response to treatment, so was intubated and ventilated. A CT head scan was normal. Cardiac echocardiogram showed poor contractility and a small amount of tricuspid regurgitation. She was transferred to Sheffield Children's Hospital and had a cardiac arrest on route. She then suffered further multiple cardiac arrests and despite maximal attempts at resuscitation she eventually died during the course of these.

On post mortem examination there were no specific macroscopic abnormalities. Infection screen identified rotavirus in the stool. The brain showed hypoxic injury. The liver showed mild fatty change. Skeletal survey, metabolic and toxicology screens were normal. Fatty acid oxidation levels were carried out on skin fibroblasts and were normal. The heart appeared normal in size, shape and structure. Histology of the heart showed areas of recent necrosis, thought to be related to the recent cardiac arrests. There was also evidence of long-standing myocyte loss with increased interstitial collagen and focal myocyte fibre disarray in the left ventricle and interventricular septum. The disarray was considered insufficient for a diagnosis of Hypertrophic Cardiomyopathy. Tests for myocarditis were normal. CSF glucose was low, but this was performed on a post mortem sample.

Their second child (II:2) is fit and well and is now 4 years of age. A recent echocardiogram was normal, as were lactate, acylcarnitine and CK.

P9 (PPA2: c.[500C>T];[500C>T], p.[Pro167Leu];[Pro167Leu])

Their third child (II:3) suffered a viral illness at the age of 8 months and then suffered a week's history of increasing hypotonia and weakness. CK was 15,000 at this time and plasma lactate was raised at 5. Free carnitine was normal at 43.6, but propionylcarnitine raised at 2.38 (<1.5). Urine organic acids were normal. There was no involvement of respiratory muscles. Renal function was normal. Over a period of two weeks her weakness and hypotonia improved and her CK reduced. Echocardiogram at this time was normal.

She presented again at the age of 11 months to hospital with diarrhoea and vomiting, her oral intake was poor and she was not passing urine. She became increasingly drowsy and capillary refill was prolonged at 3 seconds. An initial blood gas showed pH 6.9, bicarbonate 13.8, base excess -13.1, lactate 8.7 and glucose 6.18. She then suffered a focal seizure with lateral gaze to the left and left sided upper limb jerks, which subsequently generalized and lasted for 12 minutes. She was given IV Lorazepam, a fluid bolus, IV antibiotics and acyclovir. Further seizures followed which were treated with IV Lorazepam, Phenytoin and PR Paraldehyde. At three hours following admission seizures had settled but she was still drowsy. CT head scan at this time was normal. Not long after this she

suffered a further focal seizure involving the left upper limb. She then suffered a cardiac arrest and was intubated and ventilated. Maximal CPR was continued for 20 minutes but was not successful.
Post mortem examination showed very extensive fibrosis of the heart muscle and normal appearance of the skeletal muscle. The brain looked normal at post mortem. Norovirus infection was confirmed on stool samples from admission.
Respiratory chain analysis on peripheral muscle tissue was normal, and histological and histochemical assessment of muscle biopsy did not reveal any major mitochondrial abnormalities.

Family 4 (P10)

This family lives in Northern Ireland, the parents are non-consanguineous. The first of their two children was affected by fatal childhood disease.

P10 (PPA2: c.[380G>T];[514G>A], p.[Arg127Leu];[Glu172Lys])

Individual P10 is a male and was the first child born to non-consanguineous parents. He was born by emergency caesarean section for failure to progress but was not admitted to the special care baby unit. His birth weight was 4.59 kg. He had some feeding difficulties in his first year and was on Nutramigen for possible allergies. His feeding settled and he was changed onto normal milk at 6 months of age. He had an admission at 10 months with a short seizure. This settled spontaneously and he was observed overnight.

He had a prolonged admission to PICU at 1 year of age following a coryzal illness when he developed cardiomyopathy, multiorgan failure and rhabdomyolysis requiring inotropic support and dialysis. He was intubated and ventilated for over 3 weeks. Initial metabolic investigations suggested an underlying VLCAD deficiency (his acylcarnitine profile was abnormal with elevated C14:1, C14 and C16:1 suggestive of VLCAD, MIM: 609575), but excluded on fatty acid oxidation studies from Sheffield. CT brain was normal. ECHO showed markedly dilated left ventricle with moderate decrease in left ventricular function. Viral myocarditis was considered, but no virus was isolated. His urinary organic acids showed large increase in 3 hydroxybutyrate and acetoacetate. Plasma amino acids were essentially normal.
Exome sequencing revealed a heterozygous mutation in *ACADVL* (NM_000018.3): c.1844G>A, p.(Arg615Gln), which is a variant of unclear clinical relevance. In the ExAC consortium this mutation is found in 345 of 121088 alleles (allele frequency 0.002849) in heterozygous state and two individuals are homozygous in this collective. Minimal coverage of *ACADVL* was 11-fold in exome analysis.

On transfer to the wards they had concerns about his neurocognitive state. MRI brain showed no structural abnormality but mild enlargement of the ventricular system in keeping with an atrophic process. MR spectroscopy was normal. He had a normal eye examination. Brain stem auditory evoked responses were normal. He had intensive physiotherapy and made a good recovery, but had some central weakness. He was discharged from hospital at 14.5 months (admitted for 2.5 months). Repeat ECHO prior to discharge showed normal left ventricular function with a degree of muscle thickening.

His health was reasonably good and he attended the Child developmental clinic and he appeared to show some regression in terms of communication and social interaction, and had bilateral alternating squint. Paediatric cardiology review and echocardiogram at 18 months showed good systolic function with an ejection fraction of 74% and mild left ventricular hypertrophy.

At 2 years of age, he was admitted with vomiting and diarrhoea secondary to Norovirus infection. He deteriorated over the course of a day and developed a mixed metabolic and respiratory acidosis and afebrile seizures. He was transferred to PICU and unfortunately had an asystolic cardiorespiratory arrest with no response to resuscitation.

Respiratory chain analysis on peripheral skeletal muscle showed no evidence of a mitochondrial respiratory chain defect; muscle biopsy showed no evidence of myopathic or neurogenic disorder, no fibre variation, necrosis, inclusions, ragged-red or cytochrome c oxidase-deficient fibres.

A metabolic post-mortem was performed (Figure S1). Cause of death was due to cardiac failure secondary to myocardial fibrosis and acute myocardial ischaemia due to mitochondrial myopathy. The post-mortem heart was enlarged (86 g in weight) with evidence of mild hypertrophy of the left ventricular wall which was 1.0 cm in thickness. There was transmural irregular pallor and fibrosis on sectioning and the myocardium was stiff and dense in texture although no evidence of endocardial fibroelastosis on naked eye examination. Routine histology revealed bilateral pleural effusions and a pericardial effusion with pulmonary oedema. There was extensive transmural fibrosis of the left ventricle and the septum of the heart with board swathes of fibrotic tissues replacing the myocytes. Considerable myocyte nuclear pleomorphism and hyperchromasia with variation in myocyte size was noted, consistent with transmural fibrosis secondary to ischaemic injury. Endocarditis was not observed, whilst the coronary arteries showed no vasculitis or thrombosis. As documented in the main manuscript text, assessment of respiratory chain enzyme activities in a cardiac muscle sample

revealed a significant and isolated mitochondrial respiratory chain defect involving complex I in isolation.

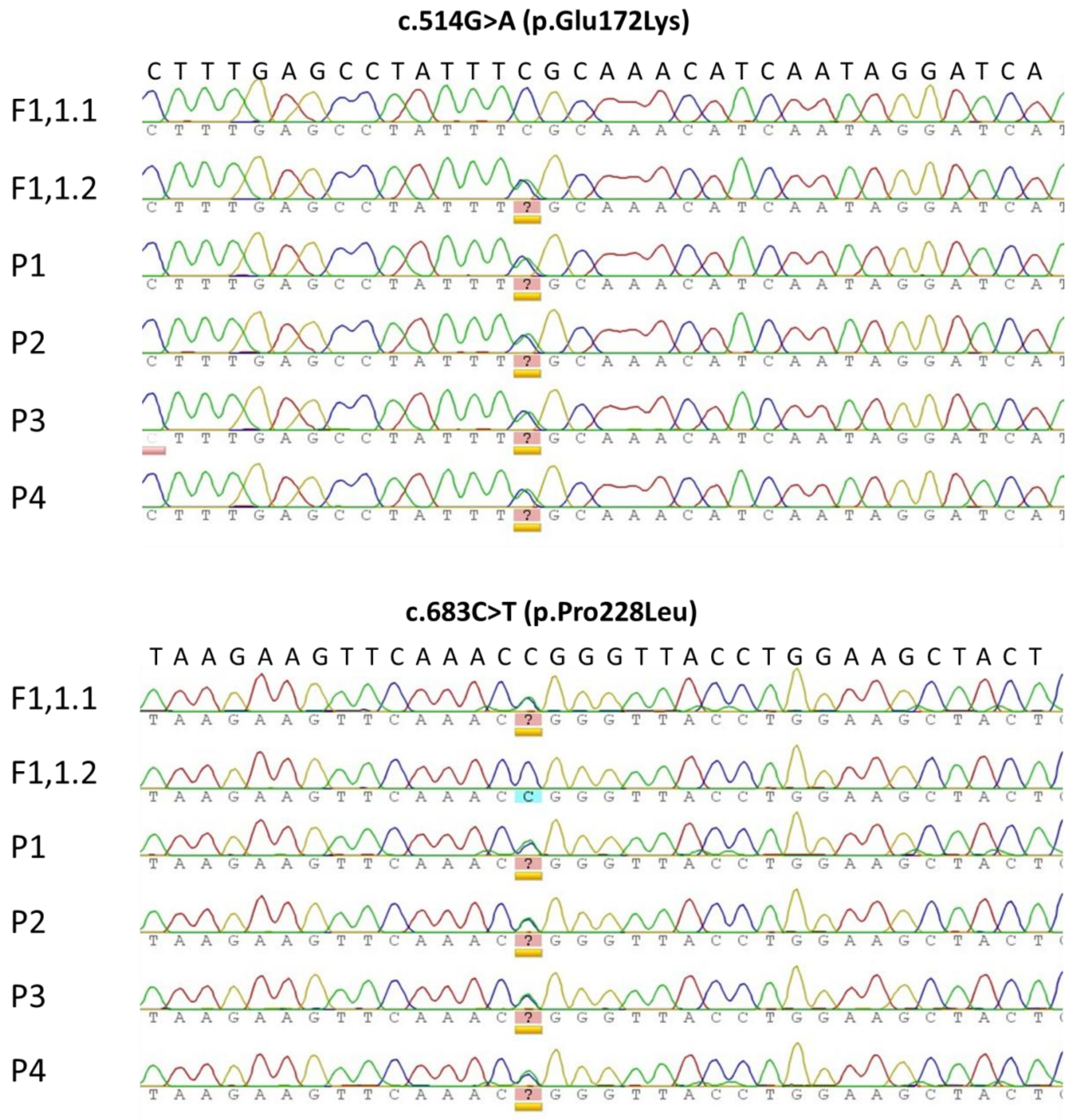

Figure S1. Sanger sequencing in Family 1 revealed compound heterozygous mutations in *PPA2* in all affected individuals.

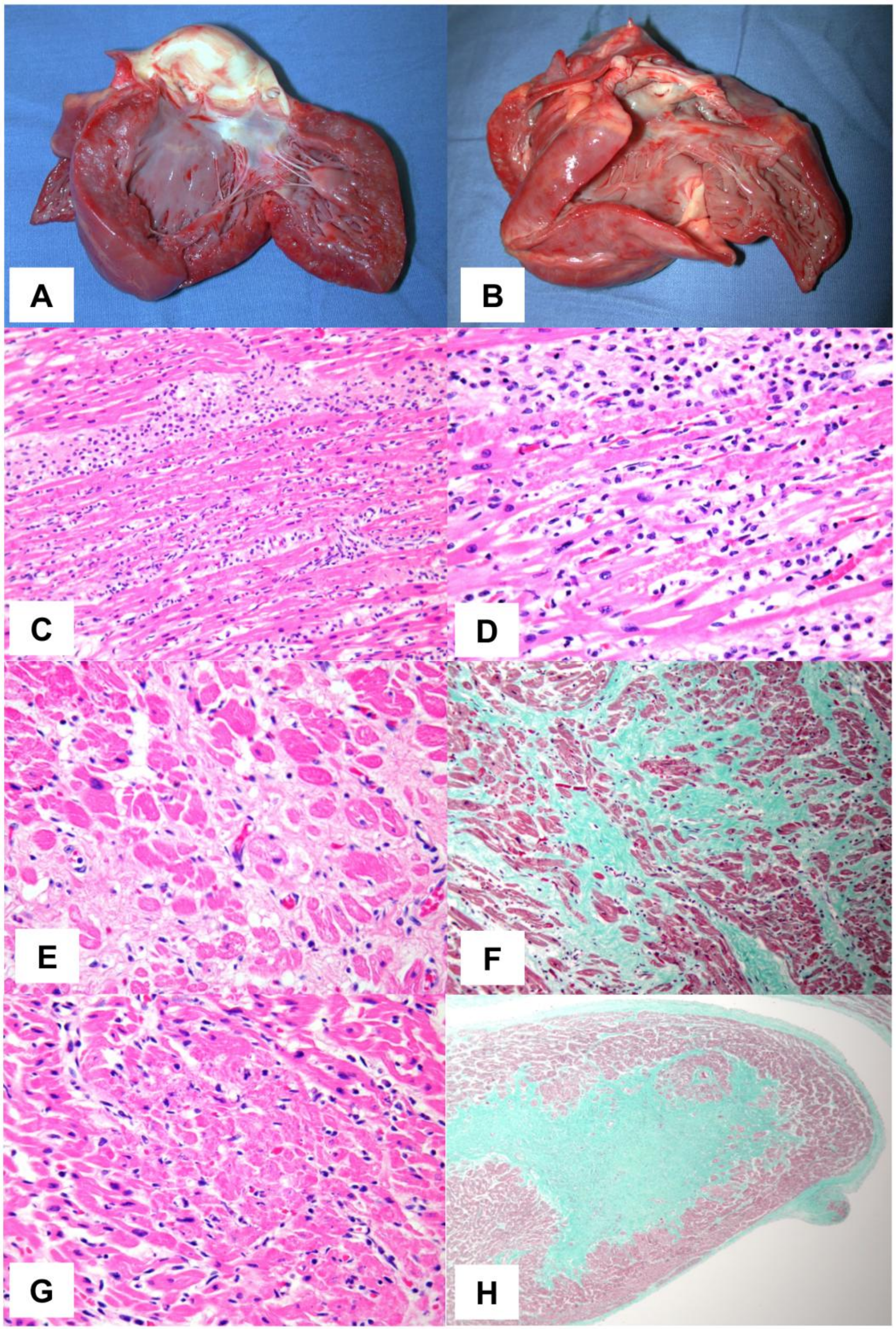

Figure S2. Post-mortem findings in the cardiac muscle from Patient 10. (A, B) Gross examination revealed an enlarged heart with evidence of left ventricular hypertrophy. The myocardium was stiff and firm, with extensive areas of pallor noted in this tissue. (C) Low and (D, E) higher power haematoxylin and eosin (H&E) staining revealed areas of acute inflammatory infiltrate between and around cardiac muscle cells, with evidence of acute myocyte necrosis (panels C and D) as well as evidence of older degenerative changes including fibrosis and nuclear pleomorphism (E). Masson trichrome staining confirms extensive fibrosis, with areas of cardiac muscle (staining red-purple color) replaced by collagenous fibrotic tissue (green-teal color) (F). Additional pathological findings include areas of early ischaemic necrosis that can be easily differentiated from the less-visibly damaged cardiac muscle cells around it (G) and severe fibrosis of a papillary muscle (Masson trichrome stain) which might be implicated in the observed valvular dysfunction (H).

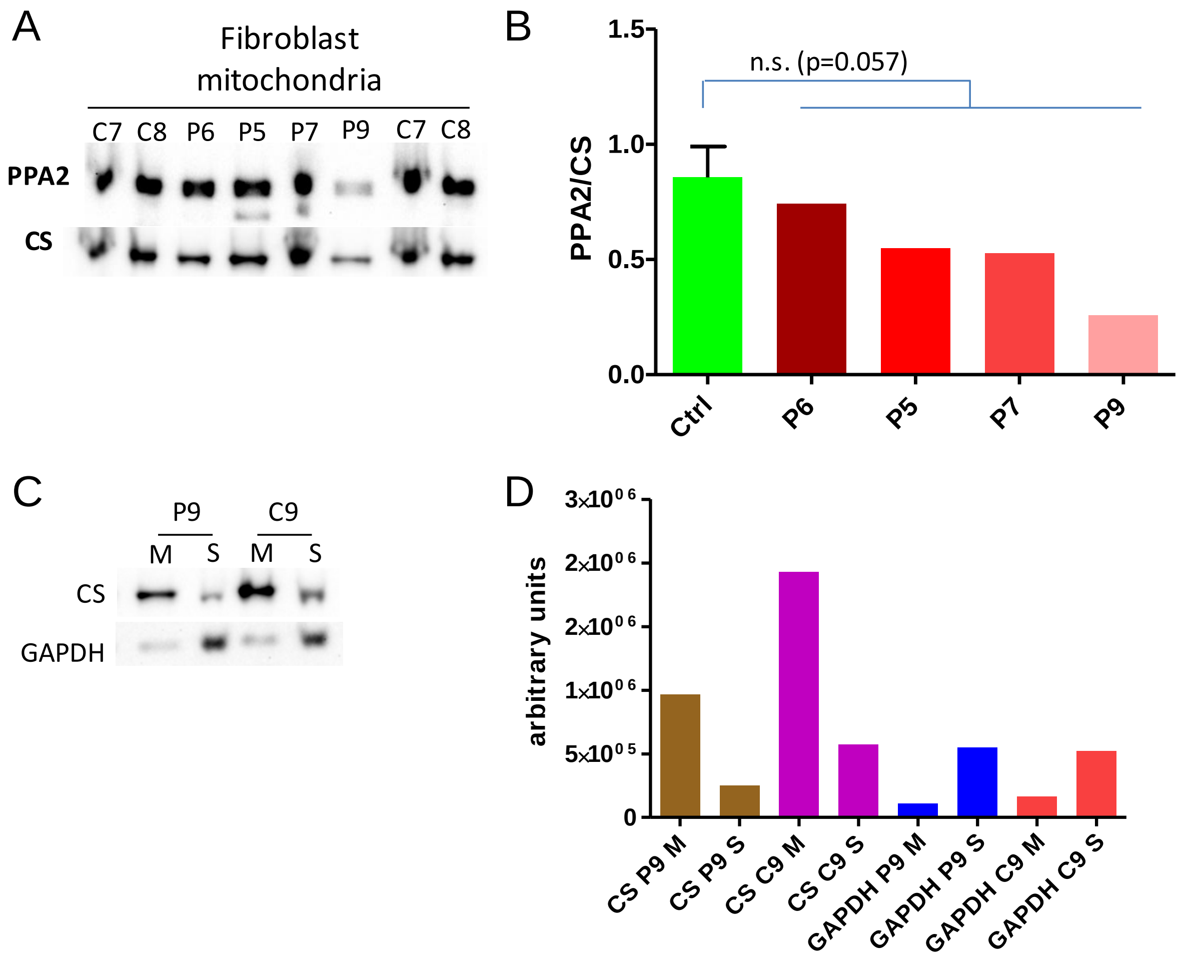

Figure S3. Western blot analysis from mitochondria isolated from fibroblasts. Isolated mitochondria form fibroblasts were available from affected individuals P5, P6, P7 and P9. Antibodies against PPA2 and citrate synthase (CS) were used (A-B). Antibodies against citrate synthase and glyceraldehyde 3-phosphate dehydrogenase (GAPDH), cytosolic housekeeping protein, were used (C-D). The supernatant (S) of mitochondria (M) isolation was investigated in individual P9, which showed only small amounts of cross contaminations of mitochondria with cytosolic protein (C-D). Statistical analysis was performed by Student's unpaired t-test. The error bar indicates the standard error of the mean (B). Abbreviation: n.s., not significant.

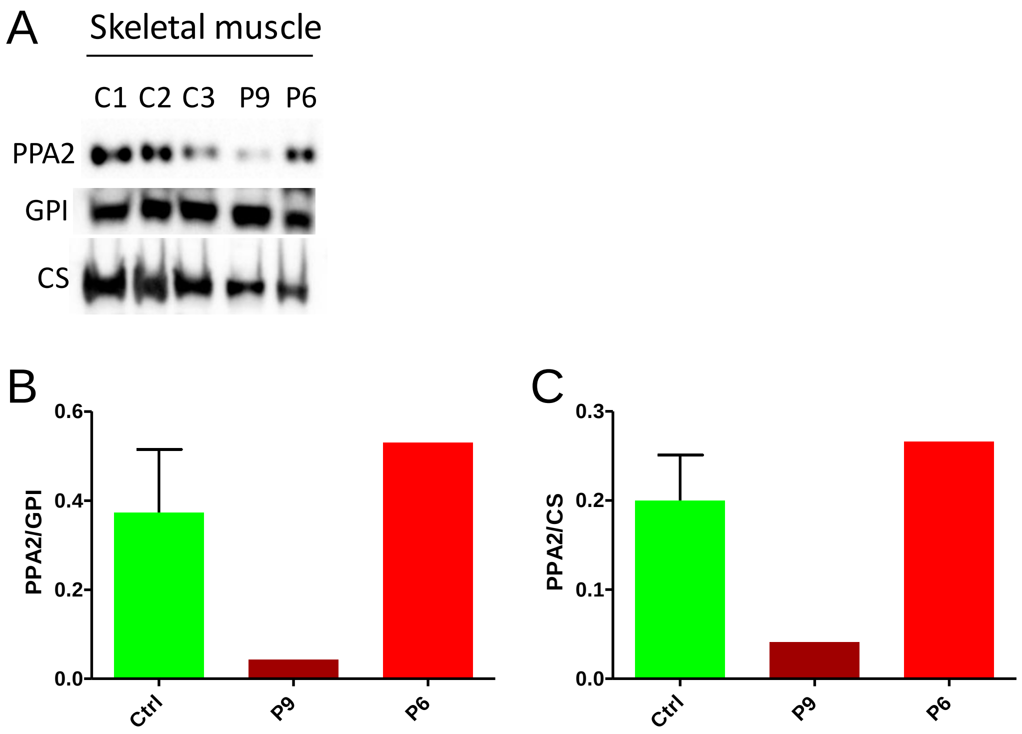

Figure S4. Western blot analysis from 600 g supernatants of skeletal muscle. Frozen skeletal muscle autopsy samples were available from affected individuals P6 and from P9. Antibodies against PPA2, citrate synthase (CS), mitochondrial matrix protein, and glucose-6-phosphate isomerise (GPI), cytosolic housekeeping protein, were used (A). Relative ratios of PPA2/GPI (B) and PPA2/CS (C) were quantified. The error bars in this graph indicate the standard error of the mean.

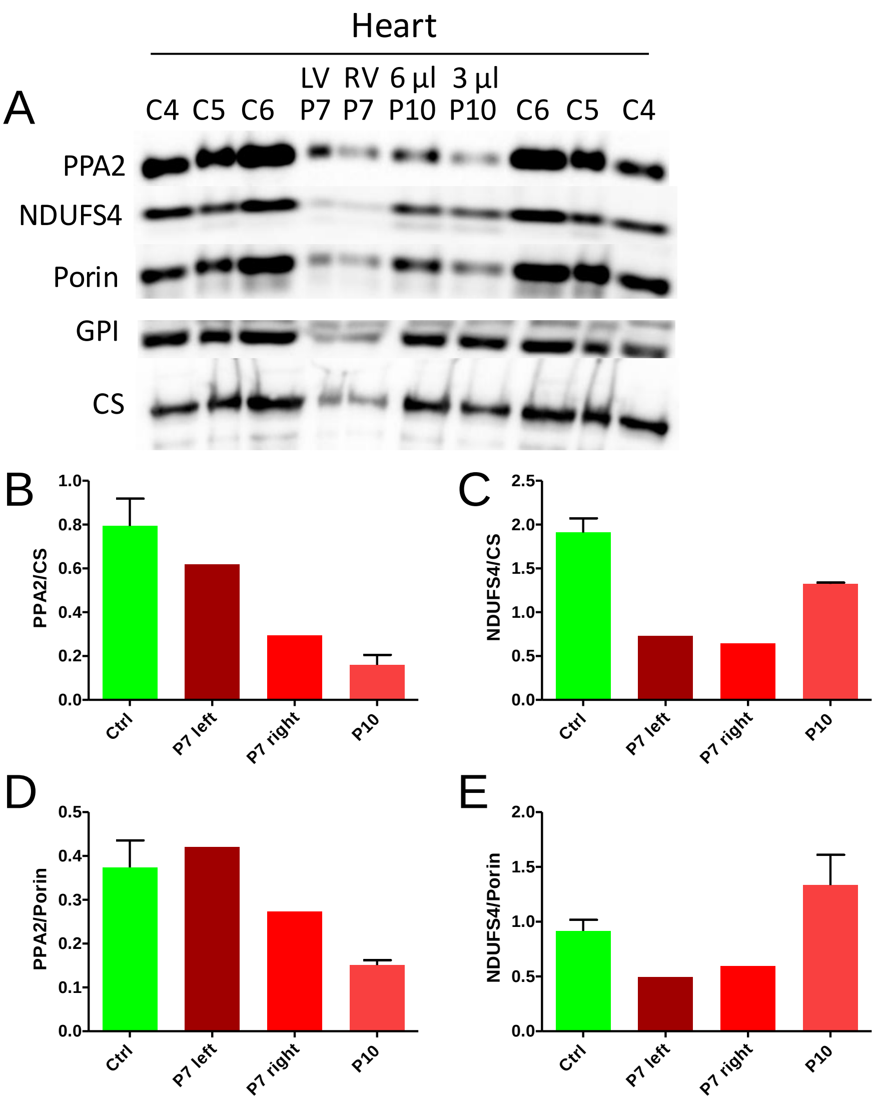

Figure S5. Western blot analysis from 600 g supernatants of heart homogenates. Frozen heart autopsy samples were available from affected individuals P7, left (LV) and right ventricle (RV), and from P10. Antibodies against PPA2, NDUFS4, subunit of complex I, citrate synthase (CS), mitochondrial matrix protein, porin, mitochondrial outer membrane protein, glucose-6-phosphate isomerise (GPI), cytosolic housekeeping protein, were used (A). Relative ratios of PPA2/CS (B), NDUFS4/CS (C), PPA2/porin (D), and NDUFS4/porin (E) were quantified. The error bars in this graph indicate the standard error of the mean.

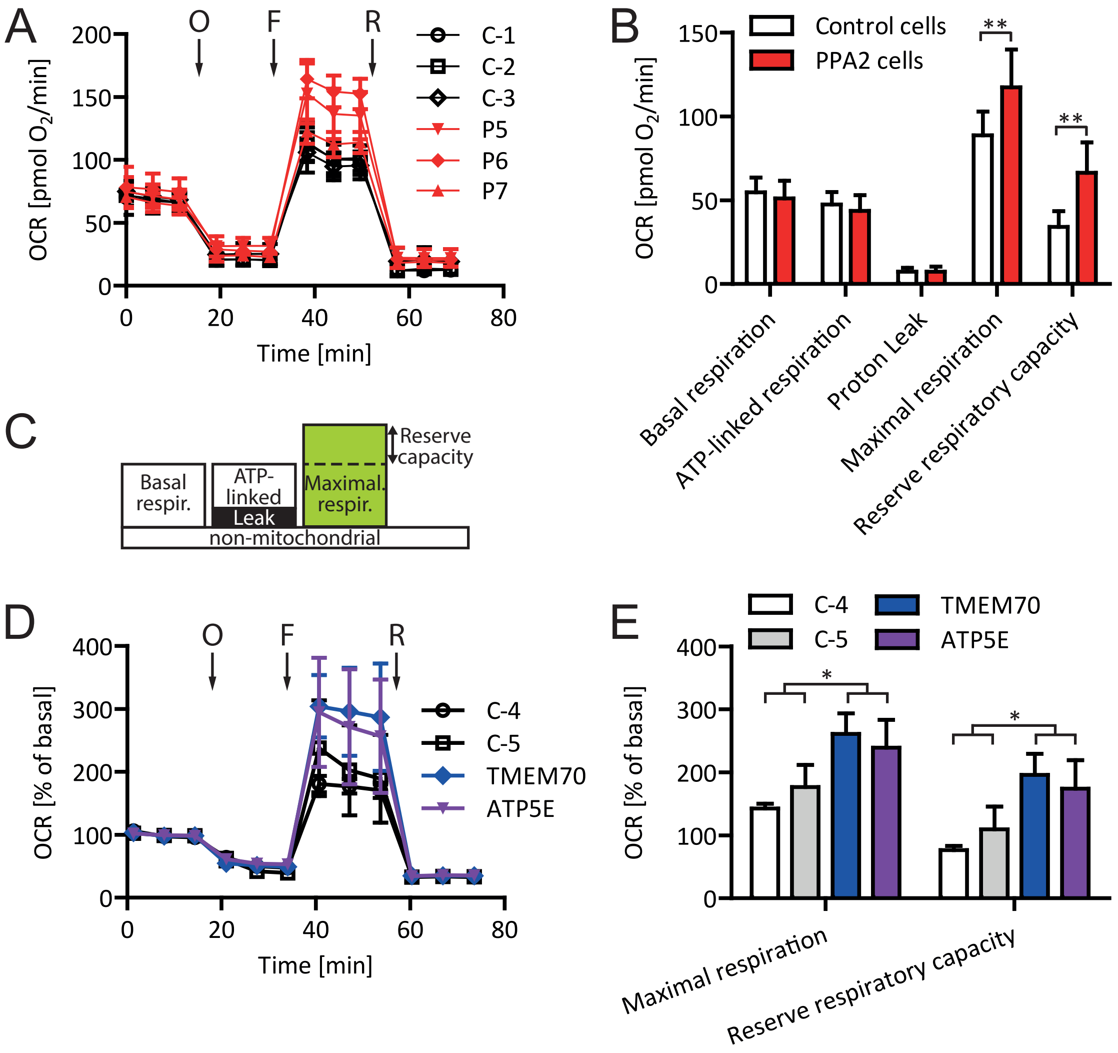

Figure S6. Oxygen consumption rates of PPA2 deficient fibroblasts. (A) Oxygen consumption rates (OCR) have been determined in three control (C-1 to C-3) and three affected with PPA2 mutations (P5, P6, and P7) and revealed increased maximal respiration as well as reserve respiratory capacity (B, C). Fibroblasts from individuals with proven ATP synthase deficiency and mutations in either TMEM70 and ATP5E showed a similar result with increased reserve respiratory capacity (D, E). Oligomycin (O, 1.0 µmol/l), carbonyl cyanide-4-(trifluoromethoxy)-phenylhydrazone (F, 0.4 µmol/l) and rotenone (R, 2.0 µmol/l) were added during the experiment. *P<0.01, **P<0.0001 in Student's unpaired t-test. The error bars in this graph indicate the standard error of the mean.

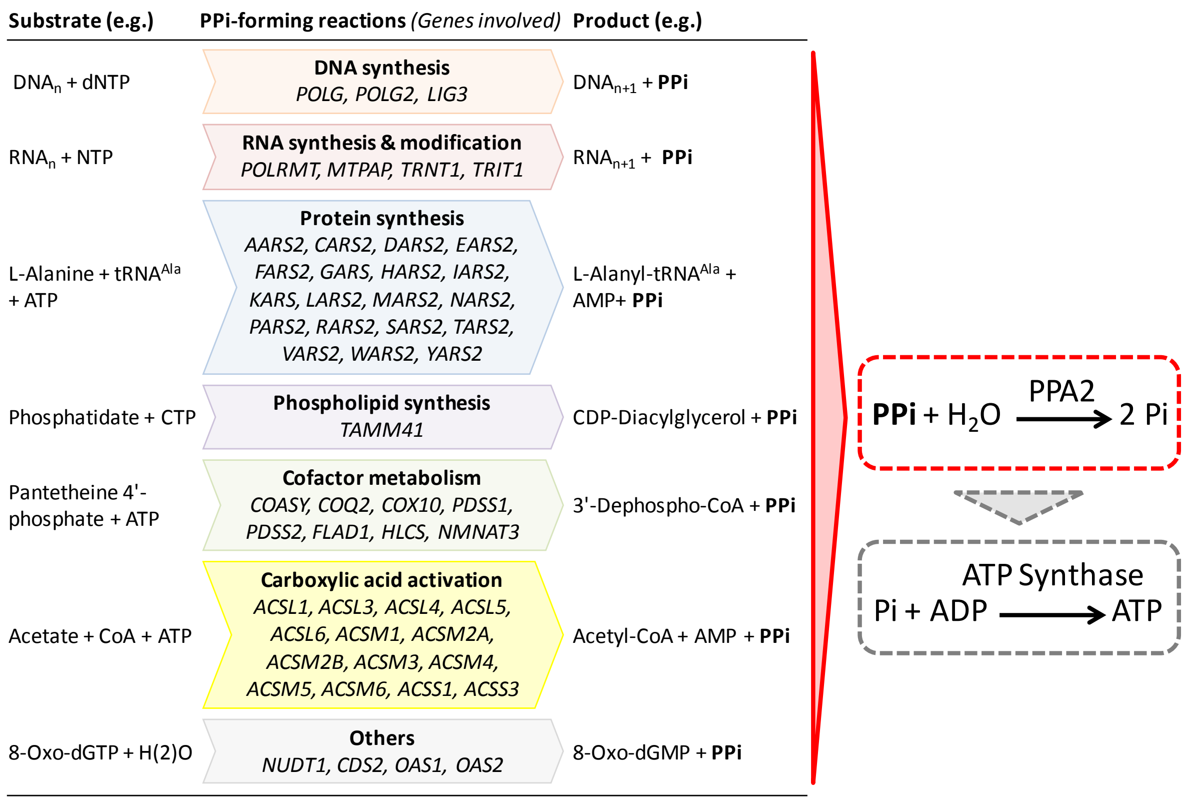

Figure S7. Reactions in Mitochondria Upstream and Downstream of Inorganic Pyrophosphate (PPi). Abbreviations: Deoxynucleotide triphosphate (dNTP), deoxynucleotide monophosphate (dAMP), nucleotide triphosphate (NTP), nucleotide monophosphate (NMP), adenosine triphosphate (ATP), adenosine diphosphate (ADP), adenosine monophosphate (AMP), cytidine triphosphate (CTP), cytidine monophosphate (CMP), Coenzyme A (CoA), deoxyguanosine triphosphate (dGTP), deoxyguanosine monophosphate (dGMP), orthophosphate (Pi), mitochondrial pyrophosphatase (PPA2).

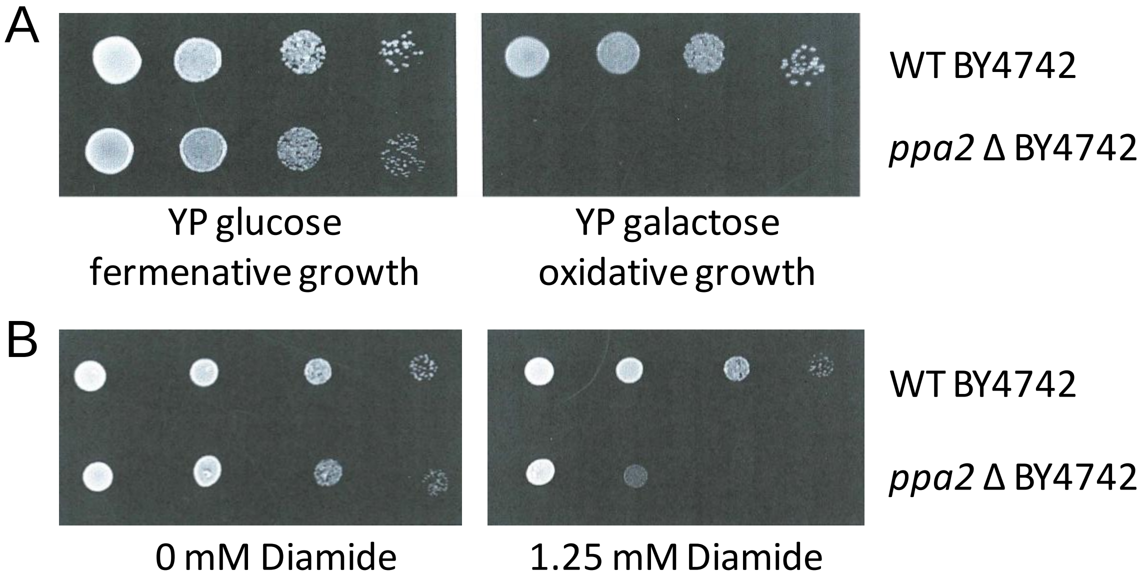

Figure S8. Investigation of yeast PPA2 knock-out cells. Growth defect of a wild type (WT BY4742) and a PPA2 knock-out (*ppa2* Δ BY4742) *S. cerevisiae* strain on aerobic medium showing that PPA2 is critical for mitochondrial respiration (A). Oxidative stress sensitivity of PPA2 knock-out *S. cerevisiae* as measured by growth defect of diamide, which oxidises intracellular thiols and mimics oxidative stress in yeast (B).

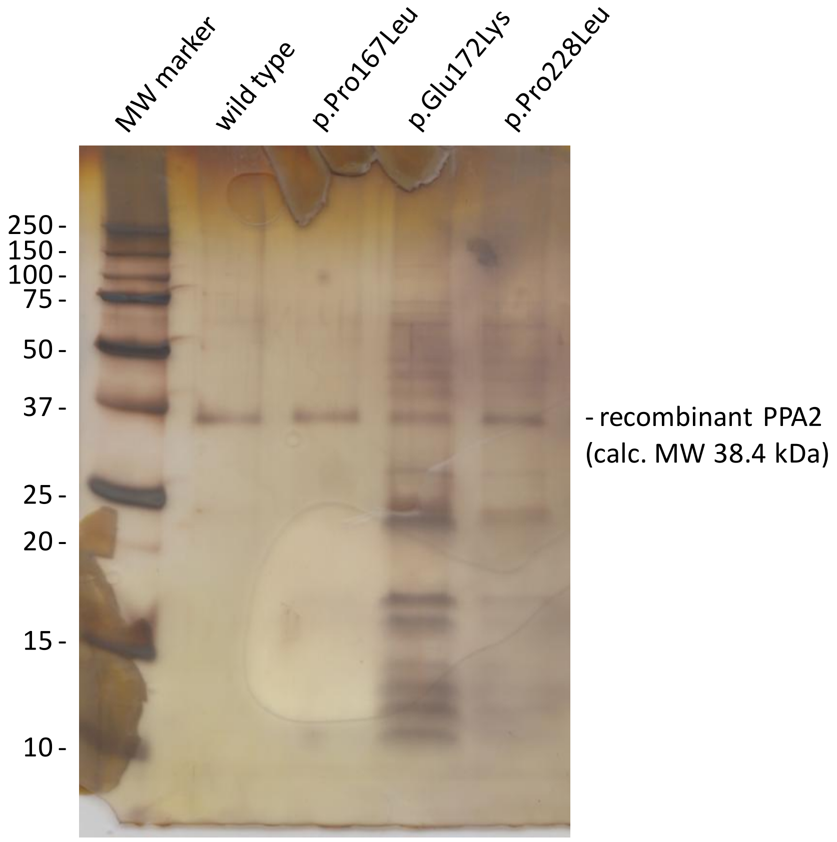

Figure S9. Silver staining of recombinant His-tagged purified PPA2 protein. The same volumes of recombinant protein as used for western blotting in Figure 3G were loaded on a 10% SDS polyacrylamide gel.

Table S1. Information on exome sequencing performed in three different centers.

Sequencing Centre:	Christchurch, NZ								
**Individual ID**	**Instrument**	**Library Prep**	**Exome Capture**	**Reads**	**Mapped**	**% Mapped**	**Mean cov**	**Cov 30x**	**Variant annotation method**
F1, 1.1	Illumina HiSeq® 2000	Illumina TruSeq® DNA LT	Illumina TruSeq® Exome Enrichment	158461290	51814344	87,15	50.15	95,15	Illumina HiSeq® analysis software enrichment pipeline v.0.9, followed by ANNOVAR and ENCODE Gencode v.12 (GRCh37). Variants were sequentially filtered against the 1000 Genomes (Apr 2012 release) and dbSNP137 databases. Variants with a minor allele frequency >0.01 were removed.
F1, 1.2	Illumina HiSeq® 2000	Illumina TruSeq® DNA LT	Illumina TruSeq® Exome Enrichment	125910016	42782905	86,58	39.75	95,33	
P2	Illumina HiSeq® 2000	Illumina TruSeq® DNA LT	Illumina TruSeq® Exome Enrichment	177926998	63271018	87,43	59,27	95,11	
P4	Illumina HiSeq® 2000	Illumina TruSeq® DNA LT	Illumina TruSeq® Exome Enrichment	147463447	43937929	87,95	42,45	94,95	

Sequencing Centre:	Munich, GER								
**Individual ID**	**Instrument**	**Library Prep**	**Exome Capture**	**Reads**	**Mapped**	**% Mapped**	**Avg cov**	**Cov 20x**	**Variant annotation method**
P6, 85154	Illumina HiSeq® 2500	SureSelect XT Target Enrichment system for Illumina	SureSelectAllExon v5	113371747	112668276	99.38	144.71	97.40	Reads were aligned to genome assembly hg19 with Burrows-Wheeler Aligner (BWA, V.0.5.87.5) and genetic variation was detected using SAMtools (V.0.1.18), PINDEL (V.0.2.4t) and ExomeDepth (V.1.0.0). Candidate genes were prioritized by searching for homozygus or potentially compound heterozygous variants with a minor allele frequency < 1% in 7,000 in-house control exomes, dbSNP, 1000 Genomes and ExAC.
P5, 85155	Illumina HiSeq® 2500	SureSelect XT Target Enrichment system for Illumina	SureSelectAllExon v5	120884660	120092778	99.34	151.17	97.74	
P7, 85152	Illumina HiSeq® 2500	SureSelect XT Target Enrichment system for Illumina	SureSelectAllExon v5	121393141	120644253	99.38	152.67	97.79	
P10, 68551	Illumina HiSeq® 2500	SureSelect XT Target Enrichment system for Illumina	SureSelectAllExon v5	103709389	102998478	99.31	129.11	97.19	

Sequencing Centre:	Leeds, UK								
**Individual ID**	**Instrument**	**Library Prep**	**Exome Capture**	**Reads**	**Mapped**	**% Mapped**	**Mean cov**	**Cov 20x**	**Variant annotation method**
P8 (JT609)	Illumina HiSeq® 2500	SureSelect XT Target Enrichment system for Illumina	SureSelectAllExon v5	72844614	71182311	97.71	73.41	90.8	In house pipeline: Alignment carried out using Novoalign. Variants were called using the HaplotypeCaller (GATK, Broad Institute) .vcf files were annotated using Ensembl's Variant Effect Predictor (VEP). Local Perl scrips were used to remove variants present at >1% minor allele frequency in the following databases: dbSNP 138 and previous, NHLBI Exome Sequencing Project
P9 (JT579)	Illumina HiSeq® 2500	SureSelect XT Target Enrichment system for Illumina	SureSelectAllExon v5	74044360	72332089	97.68	72.14	90.6	
I:1 (JT576)	Illumina HiSeq® 2500	SureSelect XT Target Enrichment system for Illumina	SureSelectAllExon v5	73769160	72104134	97.74	76.35	90.2	

I:2 (JT577)	Illumina HiSeq® 2500	SureSelect XT Target Enrichment system for Illumina	SureSelectAllExon v5	66440752	64892409	97.67	62.66	88.7	(ESP) Exome Variant Server, the Exome Aggregation Consortium (ExAC), and over 3000 ethnically-matched control samples. Variants were retained if predicted ‘pathogenic’ by any one of Polyphen2, SIFT or condel. Variants were ordered by CADD score and those with CADD score >15 were retained.
II:2 (JT578)	Illumina HiSeq® 2500	SureSelect XT Target Enrichment system for Illumina	SureSelectAllExon v5	59183468	57848931	97.75	62.23	87.7	

Table S2. Compounding missense mutations in cardiomyopathy/mitochondrial associated genes, excluded from further analysis due to non-segregation within Family 1.

GENE	VARIANT			EXAC FREQUENCY	SEGREGATION WITH PHENOTYPE?
KCNJ12	rs1657740	NM_021012.4(KCNJ12):c.353G>A	p.Arg118Gln	0.4994 (60403/120958)	NO
	rs77048459	NM_021012.4(KCNJ12):c.715G>A	p.Glu239Lys	0.2807 (27048/96360)	NO
TTN	rs56341835	NM_001267550.1(TTN):c.10213G>A	p.Glu3405Lys	0.0007518 (90/119710)	NO
	rs142094090	NM_001267550.1(TTN):c.50515G>A	p.Glu16839Lys	0.0007932 (96/121024)	NO
AARS2	rs79962181	NM_020745.3(AARS2):c.1649G>C	p.Gly550Ala	0.0007925 (96/121138)	NO
	rs142094090	NM_020745.3(AARS2):c.1621G>A	p.Glu541Lys	0.0007932 (96/121024)	NO

Table S3. Prediction Tools - Estimation of pathogenic relevance

1. Prediction for PPA2 mutations identified by exome sequencing

**Mutation**<br>**Protein**	**c.380G>T**<br>**p.Arg127Leu**	**c.500C>T**<br>**p.Pro167Leu**	**c.514G>A**<br>**p.Glu172Lys**	**c.683C>T**<br>**p.Pro228Leu**
Family	F4	F2, F3	F1	F1, F4
ExAC, heterozygotes (total alleles)	20 (121354)	3 (120914)	59 (120800)	30 (120268)
SIFT Prediction	DAMAGING	DAMAGING	DAMAGING	DAMAGING
SIFT Score (deleterous when <0.05)	0.00	0.01	0.00	0.00
PolyPhen-2 Prediction	PROBABLY DAMAGING	PROBABLY DAMAGING	PROBABLY DAMAGING	PROBABLY DAMAGING
PolyPhen-2 (score)	0.993 (sensitivity: 0.70; specificity: 0.97)	1.000 (sensitivity: 0.00; specificity: 1.00)	0.996 (sensitivity: 0.55; specificity: 0.98)	1.000 (sensitivity: 0.00; specificity: 1.00)
Mutation Taster Prediction	disease causing	disease causing	disease causing	disease causing
Mutation Taster (probability)	0.99999999999608	0.999999999999996	0.99999999999269	0.999999999999993
Mutation Taster (predicted change)	1-amino acid sequence changed, 2-protein features (might be) affected, 3-splice site changes	1-amino acid sequence changed	1-amino acid sequence changed	1-amino acid sequence changed

2. Results for homozygous missense mutation in PPA2 from the ExAC database

**Mutation**<br>**Protein**	**c.251G>A**<br>**p.Arg84Gln**	**c.727G>T**<br>**p.Val243Leu**	**c.846G>C**<br>**p.Lys282Asn**
ExAC, homozygotes (total alleles)	1 (116414)	6 (120536)	13349 (121124)
ExAC, heterozygotes (total alleles)	34 (116414)	280 (120536)	55039 (121124)
SIFT Prediction	TOLERATED	TOLERATED	TOLERATED
SIFT Score (deleterous when <0.05)	0.18	0.15	0.14
PolyPhen-2 Prediction	benign	benign	benign
PolyPhen-2 (score)	0.005 (sensitivity: 0.97; specificity: 0.74)	0.170 (sensitivity: 0.92; specificity: 0.87)	0.038 (sensitivity: 0.94; specificity: 0.82)
Mutation Taster Prediction	polymorphism	disease causing	polymorphism
Mutation Taster (probability)	0.997195759241821	0.999989907773341	0.999999999971653
Mutation Taster (predicted change)	1-amino acid sequence changed, 2-protein features (might be) affected, 3-splice site changes	1-amino acid sequence changed, 2-protein features (might be) affected, 3-splice site changes	1-amino acid sequence changed, 2-protein features (might be) affected, 3-splice site changes

Human PPA2 sequence IDs: GenBank transcript NM_176869.2; GenBank Protein NP_789845.1; UniProt Q9H2U2; Ensembl transcript ENST00000341695; Ensembl protein ENSP00000343885
