# Supplementary material for: Sudden Cardiac Death Due to Deficiency of the Mitochondrial Inorganic Pyrophosphatase PPA2
Source: Am J Hum Genet. 2016 Aug 11;99(3):674–82. doi: 10.1016/j.ajhg.2016.06.027 (PMC5011043; doi:10.1016/j.ajhg.2016.06.027)
Supplement: Document S2. Article plus Supplemental Data [file mmc2.pdf]

REPORT

Sudden Cardiac Death Due to Deficiency of the Mitochondrial Inorganic Pyrophosphatase PPA2

Hannah Kennedy,[1,2,20] Tobias B. Haack,[3,4,20] Verity Hartill,[5,20] Lavinija Mataković,[6,20] E. Regula Baumgartner,[7] Howard Potter,[1] Richard Mackay,[1] Charlotte L. Alston,[8] Siobhan O'Sullivan,[9] Robert McFarland,[8] Grainne Connolly,[10] Caroline Gannon,[11] Richard King,[1] Scott Mead,[1,22] Ian Crozier,[12] Wandy Chan,[12,13] Chris M. Florkowski,[1] Martin Sage,[14,23] Thomas Höfken,[15] Bader Alhaddad,[3,4] Laura S. Kremer,[3,4] Robert Kopajtich,[3,4] René G. Feichtinger,[6] Wolfgang Sperl,[6] Richard J. Rodenburg,[16] Jean Claude Minet,[17] Angus Dobbie,[18] Tim M. Strom,[3,4] Thomas Meitinger,[3,4,19] Peter M. George,[1,2,*] Colin A. Johnson,[5] Robert W. Taylor,[8,21] Holger Prokisch,[3,4,21] Kit Doudney,[1,2,21] and Johannes A. Mayr[6,21,*]

We have used whole-exome sequencing in ten individuals from four unrelated pedigrees to identify biallelic missense mutations in the nuclear-encoded mitochondrial inorganic pyrophosphatase (*PPA2*) that are associated with mitochondrial disease. These individuals show a range of severity, indicating that *PPA2* mutations may cause a spectrum of mitochondrial disease phenotypes. Severe symptoms include seizures, lactic acidosis, cardiac arrhythmia, and death within days of birth. In the index family, presentation was milder and manifested as cardiac fibrosis and an exquisite sensitivity to alcohol, leading to sudden arrhythmic cardiac death in the second decade of life. Comparison of normal and mutant PPA2-containing mitochondria from fibroblasts showed that the activity of inorganic pyrophosphatase was significantly reduced in affected individuals. Recombinant PPA2 enzymes modeling hypomorphic missense mutations had decreased activity that correlated with disease severity. These findings confirm the pathogenicity of *PPA2* mutations and suggest that PPA2 is a cardiomyopathy-associated protein, which has a greater physiological importance in mitochondrial function than previously recognized.

Inorganic pyrophosphate (PPi, also termed diphosphate) is formed by several important nucleotide triphosphate-dependent reactions necessary for DNA, RNA, protein, and lipid synthesis. Pyrophosphate has to be hydrolyzed to orthophosphate (Pi). An enzyme that catalyzes this reaction is termed inorganic pyrophosphatase (PPA [Enzyme Commission number EC 3.6.1.1]) and provides Pi for biomolecules via synthesis of ATP, the terminal product of cellular energy metabolism. PPAs are found in all kingdoms of life. Type I enzymes present in *Escherichia coli* and eukaryotes depend on divalent ions, preferably Mg^{2+} ions.[1] Humans, similar to the yeast *Saccharomyces cerevisiae*, have two PPAs that share sequence homology. These comprise a cytoplasmic soluble PPA1 and a mitochondrial-located PPA2. For the latter it has been proposed that the soluble catalytic part binds to a yet uncharacterized inner mitochondrial membrane protein.[2] Knockout of the cytoplasmic *PPA1* (MIM: 179030) ortholog *IPP1* leads to a loss of viability in yeast.[3,4] Knockout of the mitochondrial *PPA2* results in a growth defect on non-fermentable carbon sources and loss of mitochondrial DNA in *S. cerevisiae*.[3]

We have identified hypomorphic missense mutations in the human gene of the mitochondrial inorganic pyrophosphatase encoded by *PPA2* (MIM: 609988) in a multicenter study by exploring undiagnosed cases with presumed mitochondrial disease using whole-exome sequencing (WES). In agreement with the Declaration of Helsinki, informed consent for genetic and biochemical studies was obtained from all study participants or their guardians.

[1]Molecular Pathology Laboratory, Canterbury Health Laboratories, Canterbury District Health Board, Christchurch 8140, New Zealand; [2]Department of Pathology, University of Otago, Christchurch 8140, New Zealand; [3]Institute of Human Genetics, Helmholtz Zentrum München – German Research Center for Environmental Health, 85764 Neuherberg, Germany; [4]Institute of Human Genetics, Technische Universität München, 81675 Munich, Germany; [5]Section of Ophthalmology & Neurosciences, Leeds Institute of Biomedical and Clinical Sciences, University of Leeds, Leeds LS9 7TF, UK; [6]Department of Pediatrics, Paracelsus Medical University Salzburg, 5020 Salzburg, Austria; [7]Metabolic Unit, University Children's Hospital Basel (UKBB), 4056 Basel, Switzerland; [8]Wellcome Trust Centre for Mitochondrial Research, Institute of Neuroscience, Newcastle University, Newcastle upon Tyne NE2 4HH, UK; [9]Department of Metabolic Paediatrics, Royal Hospital for Sick Children, Belfast BT12 6BA, UK; [10]Department of Clinical Biochemistry, Royal Victoria Hospital, Belfast BT12 6BA, UK; [11]Department of Pathology, Royal Victoria Hospital, Belfast BT12 6BA, UK; [12]Department of Cardiology, Christchurch Hospital, Canterbury District Health Board, Christchurch 8140, New Zealand; [13]University of Queensland School of Medicine, Brisbane, QLD 4006, Australia; [14]Department of Anatomical Pathology, Christchurch Hospital, Canterbury District Health Board, Christchurch 8140, New Zealand; [15]Department of Life Sciences, Brunel University London, Uxbridge, Middlesex UB8 3PH, UK; [16]Department of Pediatrics, Nijmegen Center for Mitochondrial Disorders, Radboud University Medical Centre, 6500HB Nijmegen, the Netherlands; [17]Department of Neonatology UKBB Bruderholz, University Children's Hospital Basel, 4056 Basel, Switzerland; [18]Yorkshire Regional Genetics Service, Chapel Allerton Hospital, Leeds LS7 4SA, UK; [19]DZHK (German Centre for Cardiovascular Research), partner site Munich Heart Alliance, 80802 Munich, Germany
[20]These authors contributed equally to this work
[21]These authors contributed equally to this work
[22]Present address: SEALS Genetics Laboratory, Prince of Wales Hospital, Sydney, NSW 2031, Australia
[23]Present address: New Zealand National Forensic Pathology Service, Christchurch 8013, New Zealand
*Correspondence: peter.george@cdhb.health.nz (P.M.G.), h.mayr@salk.at (J.A.M.)
http://dx.doi.org/10.1016/j.ajhg.2016.06.027.
© 2016 The Author(s). This is an open access article under the CC BY license (http://creativecommons.org/licenses/by/4.0/).

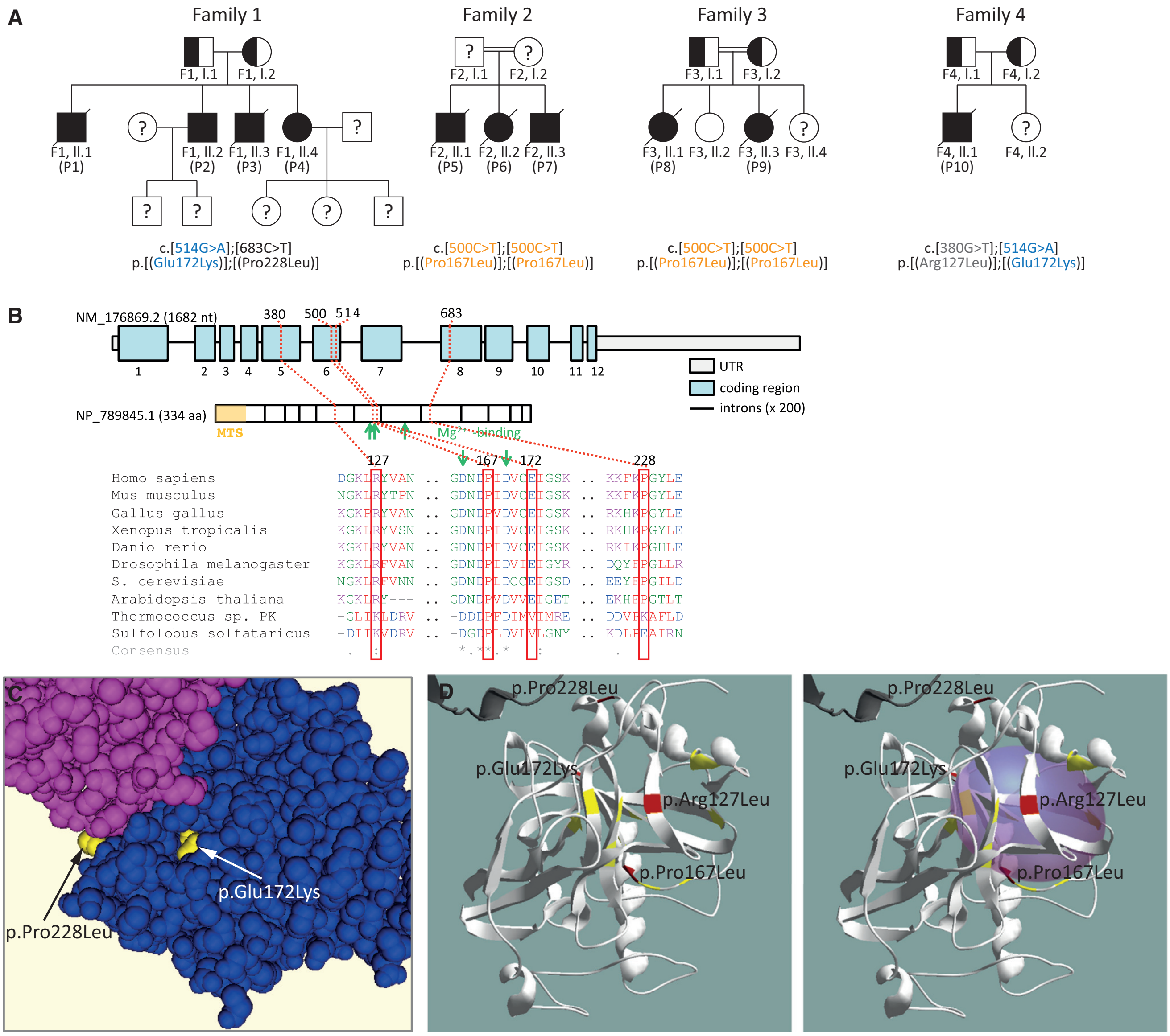

Figure 1. Pedigree Structure, *PPA2* Genomic Organization Conservation, and Family 1 Variant Modeling
(A) Pedigrees of four families identified with mutations in *PPA2* (GenBank: NM_176869.2) encoding the mitochondrial inorganic pyrophosphatase. Individuals with a question mark have not been tested. Mutations found in more than one family are colored.
(B) Location of mutations within the gene, and phylogenetic conservation of the predicted missense mutations.
(C) Space fill model showing position of p.Pro228 at boundary of dimers and p.Glu172 in the active site produced in CN3D with reference PDB: 1M38.
(D) Left: Structural model of one molecule of PPA2 showing the position of four mutations in folded structure (red). Residues that are known to be critical to PPA2 function in *S. cerevisiae* are highlighted in yellow.[19] Right: Space fill of the PPA2 active site showing three substitutions are located at the surface of the active site. Models produced using Swiss-PdbViewer[20] (with reference PDB: 1M38).

The study was approved by the Ethics Committee of the Technische Universität München and South Yorkshire Research Ethics Committee.

Family 1 consists of four affected individuals (P1–P4) born to healthy unrelated parents of European descent from New Zealand (Figure 1). This family was identified after the sudden death of two children. The first child, P1, was well until the age of 15 years when he collapsed and died after ingestion of a small volume of beer. He had no prior cardiac symptoms, but had exhibited exquisite sensitivity to alcohol in medicine and food, which was common to all four siblings in childhood. This was manifest by pallor and severe chest and arm pain after consumption of small amounts of alcohol (<0.1 g ethanol). The only abnormalities observed post mortem were in the heart, with both ventricles found to be slightly dilated. A diagnosis of myocarditis and sudden arrhythmic cardiac death was made. Individual P3 died suddenly at 20 years of age after ingestion of a small amount of alcohol (approx. 10 g ethanol). He was previously well and had no prior cardiac symptoms, but had also been exquisitely sensitive to alcohol. At post mortem

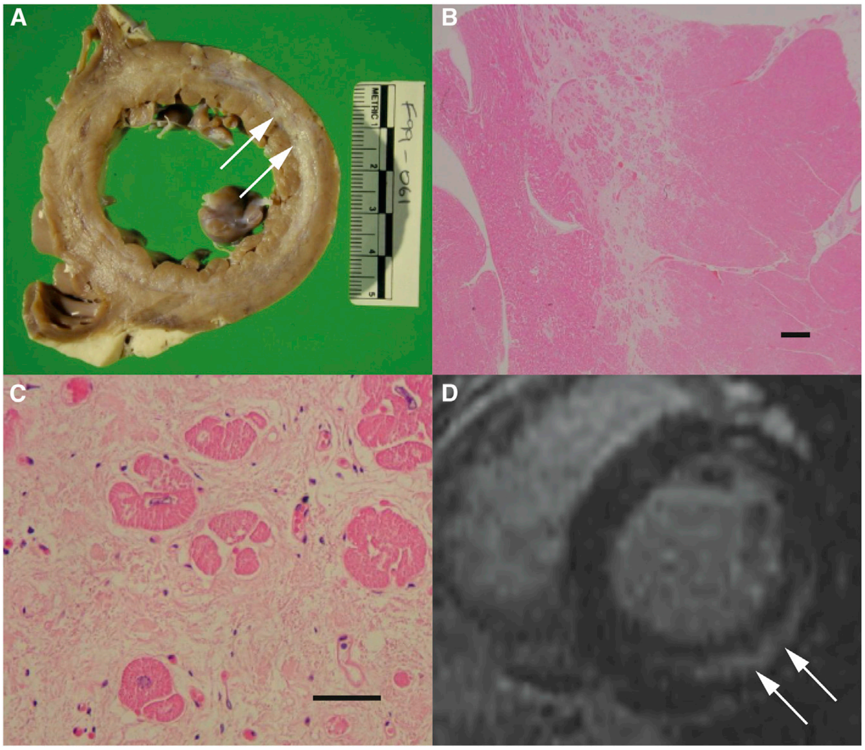

Figure 2. Cardiac Fibrosis in PPA2 Deficiency
(A) Affected individual P3, post mortem section through left ventricle showing a virtually circumferential lamina of scarring in midmyocardium with focal subendocardial involvement. Fibrosis is marked by arrows.
(B and C) Low-power (B) (bar equals 1 mm) and high-power (C) (bar equals 25 μm) microscopy of the posterior freewall of the left ventricle showing prominent mid-myocardial loose fibrosis in P3 (hematoxylin and eosin staining).
(D) Cardiac MRI showing prominent mid-myocardial fibrosis in affected individual P4 (at 25 years of age), marked by arrows.

examination the heart weighed 395 g (normal 300 g). The left ventricle was dilated with a virtually circumferential lamina of scarring in mid-myocardium. Microscopic examination revealed very widespread, mostly mature scarring of mid-myocardium in all sectors (Figure 2). Two living siblings, P2 and P4 (currently 38 and 34 years of age, respectively) were assessed based on their family history and their sensitivity to alcohol. Cardiac MRI showed marked mid-myocardial fibrosis in both siblings (P4 shown in Figure 2). They subsequently each received an implantable defibrillator for primary prophylaxis of sudden arrhythmic cardiac death, although no events have occurred to date. Despite extensive investigations into the cause of sudden death in this family over a period of >20 years, no definitive diagnosis was made (for more clinical details see Supplemental Data).

Whole-exome sequencing (see Table S1 for details) was performed on the two living siblings to elucidate the underlying molecular defect. Given that both parents appeared unaffected, we searched for rare non-synonymous variants common to the two affected children in a recessive disease model of inheritance. Four candidate genes were identified with compounding missense mutations and with an association to cardiomyopathy and/or mitochondrial function: *KCNJ12* (MIM: 602323), *TTN* (MIM: 188840), *AARS2* (MIM: 612035), and *PPA2*. Of these four genes, variants in all but *PPA2* were excluded based on non-segregation with disease (Table S2). Both affected children were compound heterozygous for *PPA2* mutations c.[514G>A];[683C>T] causing the predicted coding changes p.[Glu172Lys];[Pro228Leu], with each parent carrying one mutation. Sanger sequencing confirmed compound heterozygosity of *PPA2* mutations in the two deceased individuals, establishing the same genotype for all four affected individuals (Figure S1).

We considered that PPA2 dysfunction was the likely underlying cause of sudden cardiac death in our index family. We identified an additional three families with a further six affected individuals harboring compound heterozygous or homozygous *PPA2* mutations (Figure 1) in large exome datasets from individuals suspected of a disorder in mitochondrial energy metabolism. Family 2 (c.[500C>T];[500C>T], p.[Pro167Leu];[Pro167Leu]) (GenBank: NM_176869.2) comprises three affected siblings born to consanguineous parents from Sri Lanka. Family 3 (c.[500C>T];[500C>T], p.[Pro167Leu];[Pro167Leu]) consists of two affected and two healthy siblings born to consanguineous parents of Pakistani origin. Family 4 (c.[380G>T];[514G>A], p.[Arg127Leu];[Glu172Lys]) has one affected and one healthy sibling born to unrelated healthy parents from the United Kingdom.

Unlike the individuals from family 1, all the affected individuals in these three families presented with classical mitochondrial disease symptoms and died within the first 2 years of life of cardiac failure (Table 1). The identification of these individuals suggests that a spectrum of severity is conferred by different biallelic *PPA2* mutations. In affected individuals homozygous for c.500C>T (p.Pro167Leu), the clinical presentation involved lactic acidosis, seizures, hypotonia, and cardiac arrhythmia within the first months of life. Myocyte loss, disarray, or fibrosis was present in all individuals. Respiratory chain function varied from normal to moderate reduction in complex I and IV activities in cardiac tissue and was normal in fibroblasts and skeletal muscle tissue. The individual harboring compound heterozygous c.[380G>T];[514G>A], p.[Arg127Leu];[Glu172Lys] mutations first presented with short seizures at 10 months and developed dilated cardiomyopathy and multiorgan

Table 1. Genetic and Clinical Findings in Individuals with *PPA2* Variants

ID	Sex (M/F)	*PPA2* Variants: cDNA (NM_176869.2), Protein (NP_789845.1)	OXPHOS Activities (Normal Ranges in Brackets)	Clinical Features			Other Findings
				AO	Age at Death	Cardiac Phenotype	
F1, II:1, P1	M	c.[514G>A];[683C>T], p.[Glu172Lys];[Pro228Leu]	ND	4 years	15 years	autopsy: slight dilation of both ventricles; small pale area in the epicardium of the left ventricle, evidence of focal inflammation with neutrophils, lymphocytes, and eosinophils	sensitive to small amounts of alcohol
F1, II:2, P2	M	c.[514G>A];[683C>T], p.[Glu172Lys];[Pro228Leu]	ND	14 years	alive at 38 years	cardiac MRI: myocardial fibrosis; received implantable defibrillator	sensitive to small amounts of alcohol
F1, II:3, P3	M	c.[514G>A];[683C>T], p.[Glu172Lys];[Pro228Leu]	ND	10 years	20 years	autopsy: dilation of the left ventricle, circumferential lamina of scarring in midmyocardium with focal subendocardial involvement; very widespread mostly mature scarring of midmyocardium in all sectors	sensitive to small amounts of alcohol
F1, II:4, P4	F	c.[514G>A];[683C>T], p.[Glu172Lys];[Pro228Leu]	normal in skeletal muscle	9 years	alive at 33 years	cardiac MRI: myocardial fibrosis; received implantable defibrillator.	sensitive to small amounts of alcohol; immunohistochemical studies of skeletal muscle showed changes suggestive of a mild chronic myopathy
F2, II:, P5	M	c.[500C>T];[500C>T], p.[Pro167Leu];[Pro167Leu]	ND	10 days	11 days	autopsy: herds of fresh necrosis mainly of the right heart and interstitial lymphocyte infiltration; electron microscopy: myocard showed mitochondria with degeneration of cristae	elevated plasma lactate levels; tachypnoea and tachycardia; tonic-clonic seizures; death after severe bradycardia
F2, II:2, P6	F	c.[500C>T];[500C>T], p.[Pro167Leu];[Pro167Leu]	normal in skeletal muscle and fibroblasts	14 days	14 days	autopsy: acute and subacute necrosis more pronounced in the right heart more severe than in the left heart; electron microscopy: myocardium showed mitochondria with degeneration of cristae like in P5	metabolic acidosis with elevated plasma lactate levels; tachypneoa; vomiting, generalized seizure; cardio-respiratory decompensation; death 6.5 hr after onset of symptoms; multiple subacute necroses in the semioval center of both cerebral hemispheres
F2, II:3, P7	M	c.[500C>T];[500C>T], p.[Pro167Leu];[Pro167Leu]	normal in skeletal muscle and fibroblasts; heart muscle: LV: CI 4.1 (5.5–51.5) and CIV 64 (73.2–516.6) decreased, RV: CI not detectable, CII 9.0 (25.8–40.7), CIV 42 (73.2-516.6)	3 days	32 days	cardiac tachyarrhythmia; ECG showed hypodynamic right ventricle; autopsy: myocardium without necrosis and inflammatory infiltrations; myocytes with reduced amount of myofibrils; region of fibrosis, partially fat tissue in the right heart	elevated plasma lactate, transaminases, lactate dehydrogenase, creatine kinase (CK), CK-MB, and troponin levels

(*Continued on next page*)

Table 1. *Continued*

ID	Sex (M/F)	*PPA2* Variants: cDNA (NM_176869.2), Protein (NP_789845.1)	OXPHOS Activities (Normal Ranges in Brackets)	Clinical Features			
				AO	Age at Death	Cardiac Phenotype	Other Findings
F3, II:1, P8	F	c.[500C>T];[500C>T], p.[Pro167Leu];[Pro167Leu]	ND	5.5 months	5.5 months	autopsy: evidence for long-standing myocyte loss, increased interstitial myocyte loss, increased interstitial collagen, focal myocyte fiber disarray in the left ventricle and interventricular septum	24 hr history of vomiting and diarrhea, 1× seizures; multiple cardiac arrests; hypoxic injury of the brain; the liver showed mild fatty change
F3, II:3, P9	F	c.[500C>T];[500C>T], p.[Pro167Leu];[Pro167Leu]	normal in skeletal muscle	8 months	11 months	autopsy: extensive fibrosis of the heart muscle	plasma lactate elevated, diarrhea, vomiting; focal seizure then generalized seizure; cardiac arrest
F4, II:1, P10	M	c.[380G>T];[514G>A], p.[Arg127Leu];[Glu172Lys]	normal in skeletal muscle; CI decreased heart muscle 0.026 (0.125 ± 0.048)	10 months	2 years	echocardiography: ejection fraction of 74%, mild left ventricular hypertrophy; autopsy: extensive transmural fibrosis of the left ventricle, acute myocardial ischemia	seizures, urinary organic acids: increased 3-hydroxybutyrate, acetoacetate, and C14:1, C14, C16:1 acylcarnitine elevation in blood.

Abbreviations are as follows: AO, age at onset; F, female; M, male; ND, not determined.

failure at 1 year, necessitating intensive care for several weeks. All affected individuals died from cardiac failure after sudden deterioration. Interestingly, both individuals from family 3 and the affected individual from family 4 had viral infections at the time of hospital admission before their final heart failure (Figure S2). In the older siblings from family 2, vomiting (among other symptoms of metabolic decompensation prior to admission) was reported, one of them having loose stools once, but viral infection was not confirmed (Table 1). Clinical case histories of all affected individuals are provided in the Supplemental Data.

Western blotting showed normal amounts of PPA2 protein in fibroblast mitochondria from individuals P5, P6, and P7 but decreased amount in P9 (Figure S3). In autopsy muscle of P9, the amount of PPA2 protein was decreased, although it appeared to be normal in P6, who carried the same *PPA2* mutation (Figure S4). In heart autopsy material from P10, we noted decreased PPA2 levels as well as decreased levels of a complex I structural protein (subunit NDUFS4), correlating with the observed decrease in complex I activity in this tissue (Figure S5). In the cardiac autopsy sample of P7, PPA2 and complex I subunit levels were decreased as was the expression of the mitochondrial marker proteins porin and citrate synthase, suggestive of a more general reduction of mitochondrial number possibly due to changes in tissue composition (Figure S5).

All four missense variants involve residues of high evolutionary conservation (Figure 1) and are predicted to have a pathogenic effect on PPA2 function in silico (SIFT, PolyPhen-2, and MutationTaster) (Table S3). The high homology between the human and yeast (*S. cerevisiae*) PPA proteins facilitated predictive modeling of these human variants based on the known yeast structure of the cytosolic/nuclear pyrophosphatase IPP1 (MMDB ID: 21720; PDB: 1M38) (Figure 1). Glutamine to lysine substitution at residue 172 is predicted to disrupt at least three hydrogen bonds between interacting protein chains near the surface of the enzyme's active site. Any disruption of the active site may impair enzymatic function of PPA2. A substitution of proline to leucine at residue 228, located on the outside surface where dimer association occurs, is also predicted to disrupt the secondary structure of PPA2. Proline is a known peptide turning point amino acid and is likely to affect the orientation of the two helices between which it lies in this enzyme. We suggest that disruption of the conformation of the outer surface may impair correct dimerization of PPA2 molecules.

All four *PPA2* mutations identified in our cohort are present in the Exome Aggregation Consortium (ExAC) database (12/2015) at a frequency < 0.005, equating to 59/60,400 individuals heterozygous for p.Glu172Lys, 30/60,134 individuals heterozygous for p.Pro228Leu, 20/60,677 individuals heterozygous for p.Arg127Leu, and 3/60,457 individuals heterozygous for p.Pro167Leu (Table S1). None of these *PPA2* variants is reported in a homozygous state in ExAC, the NHLBI Exome Sequencing Project

(ESP6500) database, or 7,000 control exomes of an in-house database (Munich). Due to the complete growth defect of yeast PPA2 knockouts on non-fermentable carbon sources, it can be speculated that biallelic loss-of-function mutations of *PPA2* are incompatible with life in humans. In total, 13 LOF variants (found in 18 alleles) are published in the ExAC database and furthermore ExAC contains 71 different missense mutations (in 237 alleles) with a SIFT score ≤ 0.05 (cut-off for mutations to be considered pathogenically relevant). The cumulative heterozygous carrier frequency of these likely pathogenic *PPA2* mutations is 0.0024, which would result in a calculated prevalence for compound heterozygous or homozygous pathogenic *PPA2* mutations of 0.58 per 100,000 (1 in 170,000).

In order to investigate effects of PPA2 deficiency on the cellular metabolism, we measured oxygen consumption rate (OCR) by micro scale respirometry (XF96 Seahorse Biosciences).[5] Basal respiration and oligomycin-inhibited OCR was similar in affected individuals (P5, P6, and P7) but after the addition of the mitochondrial uncoupler FCCP, a higher activity was observed in affected individuals compared to control subjects. The difference between basal and FCCP-stimulated OCR, termed reserve respiratory capacity (RRC), was twice as high in PPA2-deficient fibroblasts compared to controls (Figures S6A and S6B). High RRC observed in PPA2-deficient cells might be due to a limitation in ATP synthesis because of insufficient Pi supply within mitochondria (Figure S7). Actually, the investigation of cells with proven ATP synthase deficiency due to mutations in either *TMEM70*[6] (MIM: 612418) or *ATP5E*[7] (MIM: 606153) revealed a similar OCR-profile with high RRC. Because high RRC is not a specific finding, further investigations were required to demonstrate decreased ATP synthesis in PPA2 deficiency.

We next determined pyrophosphatase activity in isolated mitochondria from fibroblasts of control and affected individuals, which were prepared from 540 ccm of confluent primary fibroblasts. After harvesting by trypsinization and washing twice with phosphate-buffered saline cells, the weight of the cell pellet was determined. Cells were suspended in the 10-fold amount (e.g., 500 μL per 50 mg of cell pellet) of ice-cold, hypotonic homogenization buffer (10 mmol/L Tris [pH 7.4]) and homogenized by the use of a tight fitting Potter Elvehjem homogenizer. Immediately after homogenization, 1.5 mmol/L sucrose (20% of the homogenization volume) was added to preserve mitochondria. After centrifugation at $600 \times g$, the mitochondria containing supernatant was centrifuged at $10{,}000 \times g$ and the mitochondria-containing pellet was washed twice with SEKT buffer (250 mmol/L sucrose, 2 mmol/L EGTA, 40 mmol/L KCl, 20 mmol/L KCl [pH 7.4]). The mitochondrial pellet was finally suspended in the equal amount of SEKT buffer (50 μL per 50 mg cell pellet) and stored at −80°C prior to further investigations.[7,8] The hydrolysis of PPi and quantification of orthophosphate (Pi) formed was determined according to previously published methods[9,10] with minor modifications. The incubation buffer contained 50 mmol/L Tris ([pH 8.0] 0.1 mmol/L EGTA) and the indicated concentrations of $MgCl_2$ and PPi. The reaction was started by the addition of enzyme in a final volume of 100 μL, incubated at 37°C, and stopped by the addition of 100 μL reagent A (0.70% (w/v) ammonium heptamolybdate tetrahydrate, 1.26 mol/L H_2SO_4), developed by the addition of 40 μL reagent B (0.35% (w/v) polyvinylalcohol, 0.035% (w/v) malachite green oxalate at room temperature for 20 min. The activity of PPA was significantly decreased in isolated fibroblast mitochondria from affected individuals P5, P7, and P9 at each PPi (0.001–0.1 μmol/L) and $MgCl_2$ (0.5 or 3.0 mmol/L) concentration investigated (Figures 3A and 3B). Inactivation by $CaCl_2$ was similar in affected individuals compared to control subjects (Figure 3C). Fibroblasts from affected individuals P6 and P10 did not grow sufficiently to collect enough cells for the isolation of mitochondria and from individuals P1–P4 and P8 no fibroblasts were available.

For the expression of recombinant human PPA2, wild-type *PPA2* cDNA was cloned into the expression vector pRSET B (Invitrogen) using the cloning sites BamHI and BglII.[11] The first 96 nucleotides corresponding to the cleavable N-terminal mitochondrial targeting sequence were omitted from the construct. The c.500C>T (p.Pro167Leu), c.514G>A (p.Glu172Lys), and c.683C>T (p.Pro228Leu) variants were introduced into the wild-type *PPA2* sequence by site-directed mutagenesis using Gibson assembly (New England Biolabs) with appropriate primers for PCR amplification (Phusion, New England Biolabs) and the correct coding regions of all four constructs was confirmed by Sanger sequencing. Recombinant protein was expressed in the *Escherichia coli* strain BL21(DE3)pLysS at 37°C starting at OD_{600} of 0.2 and using 1 mmol/L IPTG for 2 hr. The bacterial suspension was harvested and sonified in homogenization buffer and the supernatant was bound to HisPur cobalt spin columns (Thermo).[11] The amount of the recombinant proteins was determined by western blotting with a human PPA2 antibody (Abcam cat# ab177935). Equal amounts of either wild-type or mutant recombinant PPA2 proteins were used for the pyrophosphatase activity assay. Compared to wild-type, the p.Pro167Leu and p.Glu172Lys variants showed 5%–10% residual activity at PPi substrate concentrations 18–500 μmol/L. The p.Pro228Leu variant had a residual activity of 24%–28% in this concentration range compared to wild-type (Figure 3D). The activities of wild-type and mutants were similarly sensitive to inhibition by Ca^{2+} (data not shown).

As previously reported, *PPA2* knockout strain from *S. cereviseae* is unable to grow on aerobic media.[3] We also detected a growth defect of *PPA2* knockout yeast on diamide-containing media, which lowers antioxidant concentrations (Figure S8).[12] These antioxidants protect the cell against reactive oxygen species that are also natural by-products of mitochondrial respiratory chain function. The increased diamide sensitivity of PPA2-deficient yeast therefore suggests reduced levels of antioxidants.

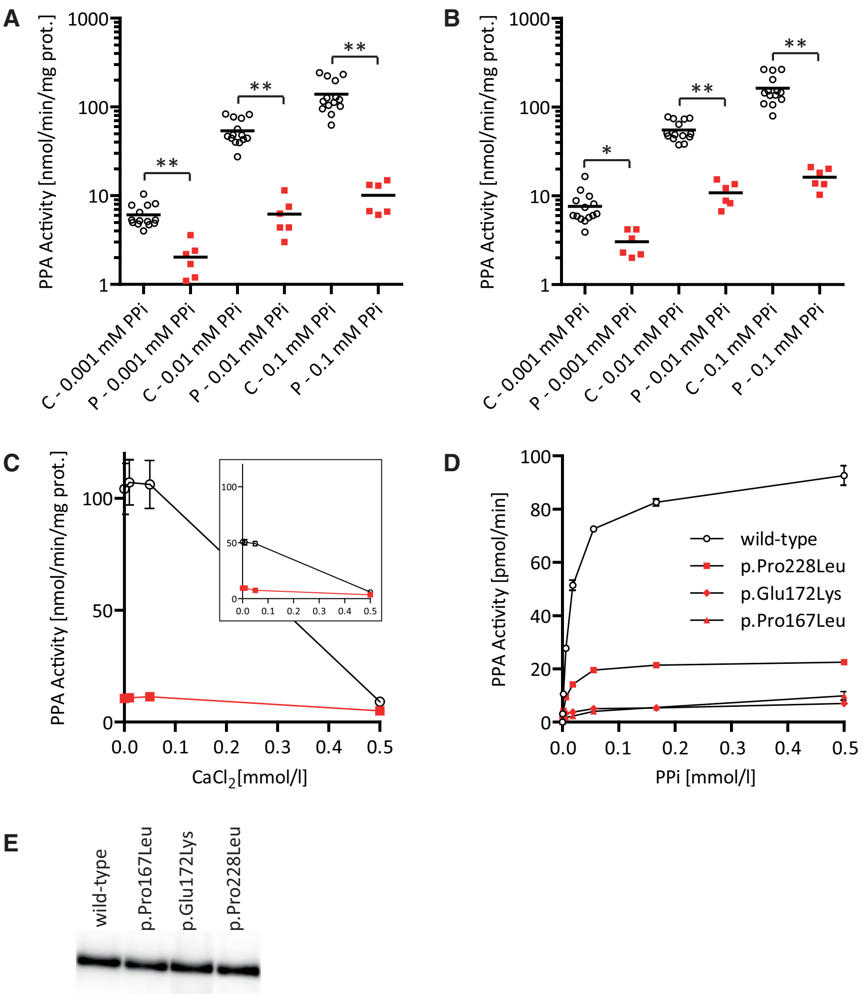

Figure 3. Inorganic Pyrophosphatase Activity in Fibroblast Mitochondria and Recombinant Enzymes

(A and B) Activity of inorganic pyrophosphatase in different fibroblast mitochondria isolations from affected individuals (P) P5, P7, and P9 compared to 14 control subjects (C) at different PPi concentrations and either (A) 0.5 mmol/L $MgCl_2$ or (B) 3.0 mmol/L $MgCl_2$.

(C) Inhibition of inorganic pyrophosphatase in fibroblast mitochondria from affected individual P5 (red squares) and three control subjects (black circles) incubated at 0.5 mmol/L $MgCl_2$ and different $CaCl_2$ concentrations and either 0.1 mmol/L PPi or 0.01 mmol/L PPi (small insert).

(D) Pyrophosphatase activity of equal amounts of recombinant proteins at different PPi concentrations.

(E) Protein amount of recombinant PPA2 protein was adjusted by western blot analysis and silver staining (Figure S9).

$*p < 0.01$, $**p < 0.0001$ in Student's unpaired t test. The error bars in this graph indicate the standard error of the mean.

with high expression in heart mitochondria.[14] In cases of severe PPA2 dysfunction, ATP depletion has an acute effect and lactic acidosis and cardiomyopathy occurs prior to chronic damage developing, which could lead to acute symptoms in the presence of secondary triggers. It is interesting to note, however, that both affected individuals in family 3 had a history of vomiting, diarrhea, and seizures prior to admission to hospital, and viral infection (rotavirus [1st] and norovirus [2nd]) was confirmed in stool samples taken at time of admission. A norovirus infection was also found in P10 from the United Kingdom. This may indicate that a viral stressor was responsible for adversely affecting mitochondrial metabolism in families 3 and 4, in the same way that alcohol was a trigger for arrhythmia in the index family. In the oldest sibling of family 2, there was also some vomiting prior to hospital admission but viral illness was not confirmed. In the two younger siblings, vomiting occurred among other initial symptoms of metabolic compensation, in the youngest sibling who was hospitalized from birth already on the third day of life. Of note, symptoms like vomiting,[15] diarrhea,[16] and seizures[17] are also typical for other disorders of the mitochondrial energy metabolism.

In the case of family 1, our data suggest that p.Pro228Leu is a relatively mild variant, given that PPA2 function is only moderately reduced. This hypothesis is supported by investigation of the activity of recombinant PPA2 enzyme activity. The p.Pro228Leu substitution resulted in a reduction of PPA activity to approximately 25% of wild-type (Figure 3). These individuals show chronic accumulation of cardiac fibrosis, and death occurred after ingestion of alcohol to which they were already known to have acute sensitivity. We propose that alcohol acted as a trigger in these case subjects, whose PPA2 dysfunction created chronic mitochondrial sensitivity and whose hearts were consistently deprived of adequate ATP resulting in fibrosis. Ingestion of alcohol appears to have increased the stress on the already sensitive mitochondria/fibrotic heart, causing cardiac arrhythmia and death. There is a link between alcohol metabolism and inorganic pyrophosphatase function that might underlie the pathology of affected individuals. Ethanol is oxidized to acetaldehyde and further to acetic acid.[13] Resulting acetic acid has to be activated to acetyl-coenzyme A, which is accompanied by the formation of equimolar amounts of PPi (Figure S7). This esterification reaction is catalyzed by short-chain acyl-CoA synthetases encoded, for example, by *ACSS1* (MIM: 614355), an enzyme

All affected individuals died from cardiac failure. Sudden, unexpected cardiac death was especially observed in P1, P3, P5, P6, and P8. As clearly seen in cardiac MRIs from the two living individuals from family 1, midmyocardial fibrosis is a pre-existing condition (Figure 2) even though no cardiac symptoms were experienced by these

individuals. Using late gadolinium enhancement (LGE), myocardial fibrosis can be clearly determined and is also a common finding in other disorders of the mitochondrial energy metabolism such as MELAS (MIM: 540000) due to the common m.3243A>G mutation of the mitochondrial DNA.[18]

In conclusion, we have identified biallelic missense mutations in *PPA2* as cause of mitochondrial cardiomyopathy and sudden cardiac death. This finding highlights a critical role of PPA2 in mitochondrial function and warrants further functional investigation. Importantly, mild mutations in *PPA2* may not have an immediate life-threatening effect until triggered by a stressor such as viral illness or alcohol metabolism, predisposing otherwise healthy individuals to sudden cardiac death. Considering the relatively high frequency of *PPA2* mutations present in the ExAC database, it is important that clinically suspicious individuals are screened for *PPA2* mutations in addition to evidence of heart fibrosis by cardiac MRI. Moreover, application of an implantable cardioverter defibrillator may prevent sudden cardiac death in at-risk individuals who harbor biallelic *PPA2* mutations.

Supplemental Data

Supplemental Information includes supplemental case reports, nine figures, and three tables and can be found with this article online at http://dx.doi.org/10.1016/j.ajhg.2016.06.027.

Acknowledgments

We thank Dr. Kym Mina and Gabe Kolle for data processing advice, Prof. Werner Wegmann (Institute of Pathology, Kantonsspital Baselland/Liestal, Switzerland) for careful autopsy work-up, and Eilidh Jackson and Ruth Charlton (Yorkshire Regional Genetics Service, Leeds Teaching Hospitals NHS Trust) for performing Sanger sequencing. This work was supported by the Maia Health Foundation, a British Heart Foundation clinical training fellowship (FS/13/32/30069 to V.H.), a Sir Jules Thorn Award (09/JTA to C.A.J.), the EC FP7-PEOPLE-ITN Mitochondrial European Educational Training (MEET) Project (GA #317433 to H. Prokisch and J.A.M.), the Add-On-Project of the PMU-FFF (A-12/01/005-SPE to W.S.), the Vereinigung zur Förderung pädiatrischer Forschung und Fortbildung, by the German Bundesministerium für Bildung und Forschung (BMBF) through the German Network for mitochondrial disorders (mitoNET 01GM1113C to T.M. and H. Prokisch), the E-Rare project GENOMIT (01GM1207 to T.M. and H. Prokisch), by the BMBF through the Juniorverbund in der Systemmedizin “mitOmics” (FKZ 01ZX1405C to T.B.H.), by the BMBF through the DZHK (German Centre for Cardiovascular Research, Z76010017300 and Z56010015300 to T.M.), and by grants to R.W.T. from The Wellcome Trust Centre for Mitochondrial Research (096919Z/11/Z), the Medical Research Council (UK) Centre for Translational Muscle Disease Research (G0601943), The Lily Foundation, and the UK NHS Highly Specialised Commissioners, which funds the “Rare Mitochondrial Disorders of Adults and Children” Diagnostic Service in Newcastle upon Tyne. C.L.A. is the recipient of a National Institute for Health Research (NIHR) doctoral fellowship (NIHR-HCS-D12-03-04). The views expressed are those of the authors and not necessarily those of the NHS, the NIHR, or the Department of Health.

Received: January 14, 2016
Accepted: June 27, 2016
Published: August 11, 2016

Web Resources

Cn3D, http://www.ncbi.nlm.nih.gov/Structure/CN3D/cn3d.shtml
Enzyme Nomenclature (Nomenclature Committee of the International Union of Biochemistry and Molecular Biology [NC-IUBMB]), http://www.chem.qmul.ac.uk/iubmb/enzyme/
ExAC Browser, http://exac.broadinstitute.org/
GenBank, http://www.ncbi.nlm.nih.gov/genbank/
MMDB, http://www.ncbi.nlm.nih.gov/structure
MutationTaster, http://www.mutationtaster.org/
NHLBI Exome Sequencing Project (ESP) Exome Variant Server, http://evs.gs.washington.edu/EVS/
OMIM, http://www.omim.org/
PolyPhen-2, http://genetics.bwh.harvard.edu/pph2/
RCSB Protein Data Bank, http://www.rcsb.org/pdb/home/home.do
SIFT, http://sift.bii.a-star.edu.sg/
Swiss PDB Viewer, http://www.expasy.org/spdbv/

References

1. Stockbridge, R.B., and Wolfenden, R. (2011). Enhancement of the rate of pyrophosphate hydrolysis by nonenzymatic catalysts and by inorganic pyrophosphatase. J. Biol. Chem. *286*, 18538–18546.
2. Volk, S.E., Baykov, A.A., Kostenko, E.B., and Avaeva, S.M. (1983). Isolation, subunit structure and localization of inorganic pyrophosphatase of heart and liver mitochondria. Biochim. Biophys. Acta *744*, 127–134.
3. Lundin, M., Baltscheffsky, H., and Ronne, H. (1991). Yeast PPA2 gene encodes a mitochondrial inorganic pyrophosphatase that is essential for mitochondrial function. J. Biol. Chem. *266*, 12168–12172.
4. Serrano-Bueno, G., Hernández, A., López-Lluch, G., Pérez-Castiñeira, J.R., Navas, P., and Serrano, A. (2013). Inorganic pyrophosphatase defects lead to cell cycle arrest and autophagic cell death through NAD+ depletion in fermenting yeast. J. Biol. Chem. *288*, 13082–13092.
5. Holzerova, E., Danhauser, K., Haack, T.B., Kremer, L.S., Melcher, M., Ingold, I., Kobayashi, S., Terrile, C., Wolf, P., Schaper, J., et al. (2016). Human thioredoxin 2 deficiency impairs mitochondrial redox homeostasis and causes early-onset neurodegeneration. Brain *139*, 346–354.
6. Cízková, A., Stránecký, V., Mayr, J.A., Tesarová, M., Havlícková, V., Paul, J., Ivánek, R., Kuss, A.W., Hansíková, H., Kaplanová, V., et al. (2008). TMEM70 mutations cause isolated ATP synthase deficiency and neonatal mitochondrial encephalocardiomyopathy. Nat. Genet. *40*, 1288–1290.
7. Mayr, J.A., Havlícková, V., Zimmermann, F., Magler, I., Kaplanová, V., Jesina, P., Pecinová, A., Nusková, H., Koch, J., Sperl, W., and Houstek, J. (2010). Mitochondrial ATP synthase deficiency due to a mutation in the ATP5E gene for the F1 epsilon subunit. Hum. Mol. Genet. *19*, 3430–3439.

8. Bentlage, H.A., Wendel, U., Schägger, H., ter Laak, H.J., Janssen, A.J., and Trijbels, J.M. (1996). Lethal infantile mitochondrial disease with isolated complex I deficiency in fibroblasts but with combined complex I and IV deficiencies in muscle. Neurology *47*, 243–248.
9. Curbo, S., Lagier-Tourenne, C., Carrozzo, R., Palenzuela, L., Lucioli, S., Hirano, M., Santorelli, F., Arenas, J., Karlsson, A., and Johansson, M. (2006). Human mitochondrial pyrophosphatase: cDNA cloning and analysis of the gene in patients with mtDNA depletion syndromes. Genomics *87*, 410–416.
10. Van Veldhoven, P.P., and Mannaerts, G.P. (1987). Inorganic and organic phosphate measurements in the nanomolar range. Anal. Biochem. *161*, 45–48.
11. Banka, S., de Goede, C., Yue, W.W., Morris, A.A., von Bremen, B., Chandler, K.E., Feichtinger, R.G., Hart, C., Khan, N., Lunzer, V., et al. (2014). Expanding the clinical and molecular spectrum of thiamine pyrophosphokinase deficiency: a treatable neurological disorder caused by TPK1 mutations. Mol. Genet. Metab. *113*, 301–306.
12. Kuge, S., and Jones, N. (1994). YAP1 dependent activation of TRX2 is essential for the response of Saccharomyces cerevisiae to oxidative stress by hydroperoxides. EMBO J. *13*, 655–664.
13. Yamashita, H., Kaneyuki, T., and Tagawa, K. (2001). Production of acetate in the liver and its utilization in peripheral tissues. Biochim. Biophys. Acta *1532*, 79–87.
14. Fujino, T., Kondo, J., Ishikawa, M., Morikawa, K., and Yamamoto, T.T. (2001). Acetyl-CoA synthetase 2, a mitochondrial matrix enzyme involved in the oxidation of acetate. J. Biol. Chem. *276*, 11420–11426.
15. Cameron, J.M., Levandovskiy, V., Mackay, N., Raiman, J., Renaud, D.L., Clarke, J.T., Feigenbaum, A., Elpeleg, O., and Robinson, B.H. (2006). Novel mutations in dihydrolipoamide dehydrogenase deficiency in two cousins with borderline-normal PDH complex activity. Am. J. Med. Genet. A. *140*, 1542–1552.
16. Rahman, S. (2013). Gastrointestinal and hepatic manifestations of mitochondrial disorders. J. Inherit. Metab. Dis. *36*, 659–673.
17. Bindoff, L.A., and Engelsen, B.A. (2012). Mitochondrial diseases and epilepsy. Epilepsia *53* (*Suppl 4*), 92–97.
18. Jose, T., Gdynia, H.J., Mahrholdt, H., Vöhringer, M., Klingel, K., Kandolf, R., Bornemann, A., and Yilmaz, A. (2011). CMR gives clue to "ragged red fibers" in the heart in a patient with mitochondrial myopathy. Int. J. Cardiol. *149*, e24–e27.
19. Heikinheimo, P., Pohjanjoki, P., Helminen, A., Tasanen, M., Cooperman, B.S., Goldman, A., Baykov, A., and Lahti, R. (1996). A site-directed mutagenesis study of *Saccharomyces cerevisiae* pyrophosphatase. Functional conservation of the active site of soluble inorganic pyrophosphatases. Eur. J. Biochem. *239*, 138–143.
20. Guex, N., and Peitsch, M.C. (1997). SWISS-MODEL and the Swiss-PdbViewer: an environment for comparative protein modeling. Electrophoresis *18*, 2714–2723.

The American Journal of Human Genetics, Volume *99*

Supplemental Data

Sudden Cardiac Death Due to Deficiency of the Mitochondrial Inorganic Pyrophosphatase PPA2

Hannah Kennedy, Tobias B. Haack, Verity Hartill, Lavinija Mataković, E. Regula Baumgartner, Howard Potter, Richard Mackay, Charlotte L. Alston, Siobhan O'Sullivan, Robert McFarland, Grainne Connolly, Caroline Gannon, Richard King, Scott Mead, Ian Crozier, Wandy Chan, Chris M. Florkowski, Martin Sage, Thomas Höfken, Bader Alhaddad, Laura S. Kremer, Robert Kopajtich, René G. Feichtinger, Wolfgang Sperl, Richard J. Rodenburg, Jean Claude Minet, Angus Dobbie, Tim M. Strom, Thomas Meitinger, Peter M. George, Colin A. Johnson, Robert W. Taylor, Holger Prokisch, Kit Doudney, and Johannes A. Mayr

Supplemental Case Reports of Four Families with *PPA2* mutations

Family 1 (P1-P4)

The family are of Caucasian origin, living in New Zealand. In the extended family there are no cases with the features seen in the family. The parents are unrelated, well and exhibit none of the features seen in their children. All 4 children are affected by the condition.

P1 (PPA2: c.[514G>A];[683C>T]; p.[Glu172Lys];[Pro228Leu])

Sibling 1; a male born in 1975 who collapsed and died suddenly in 1991 aged 15 years after drinking a small amount of beer.

He was previously well, and had no prior cardiac symptoms, but like all his siblings had been exquisitely sensitive to alcohol. This was manifest by severe chest and arm pain, and pallor following consumption of small amounts of alcohol (<0.1 g) noticed for the first time at the age of 4 years after ingestion of an alcohol-containing cough medicine. At post mortem examination the only abnormalities observed were in the heart with both ventricles being slightly dilated. A small pale area was observed on the epicardium of the left ventricle. Microscopic examination revealed evidence of focal inflammation with neutrophils, lymphocytes and eosinophils. The coronary arteries were normal. A diagnosis of myocarditis and sudden arrhythmic cardiac death was made.

P2 (PPA2: c.[514G>A];[683C>T]; p.[Glu172Lys];[Pro228Leu])

Sibling 3; a male born in 1977, was well with no cardiac symptoms, but exhibited the family sensitivity to alcohol. At the age of 14 years, a medical alcohol challenge resulted in marked pain. He was assessed because of his family history. Physical examination was normal as was ECG, echocardiogram, exercise test and Holter monitor. However an MRI scan showed marked midmyocardial fibrosis. He subsequently received an implantable defibrillator for primary prophylaxis of sudden arrhythmic cardiac death. No events have occurred to date.

P3 (PPA2: c.[514G>A];[683C>T]; p.[Glu172Lys];[Pro228Leu])

A male was born in 1978 died suddenly in 1999 aged 20 years following drinking 10 g of alcohol (one standard drink). He was previously well, and had no prior cardiac symptoms, but had also been exquisitely sensitive to alcohol noticed for the first time at the age of 10 years after accidental ingestion of a small amount of wine. At post mortem examination the only abnormalities observed were in the heart. The heart weighed 395 g (normal 300 g). The left ventricle was dilated with a virtually circumferential lamina of scarring in the midmyocardium with focal sub-endocardial involvement. Microscopic examination revealed very widespread mostly mature scarring of

midmyocardium in all sectors. No ischaemic changes were observed, nor microscopic evidence to suggest acute hypersensitivity or interstitial acute myocarditis (Figure 2). The coronary arteries were normal.

P4 (PPA2: c.[514G>A];[683C>T]; p.[Glu172Lys)];[Pro228Leu])
A female born in 1982, was well with no cardiac symptoms, but exhibited the family sensitivity to alcohol. At the age of 9 years, a medical alcohol challenge resulted in marked pain. She was assessed because of her family history. Physical examination was normal as was ECG, echocardiogram, exercise test and Holter monitor. However an MRI showed marked midmyocardial fibrosis Figure 2). She subsequently received an implantable defibrillator for primary prophylaxis of sudden arrhythmic cardiac death. No events have occurred to date.

Further investigations were undertaken on the surviving siblings to try and elucidate a shared genetic and metabolic basis for this apparently unique constellation of clinical features. Investigations have focused on possible mitochondrial genetic disorder and abnormalities of muscle structural proteins and in particular, laminopathies.

Histopathology of skeletal muscle (from P4) showed no obvious abnormality, although a muscular dystrophy or metabolic disorder could not be excluded. Stains for fat and glycogen were within normal limits. A panel of enzyme stains (including myophosphorylase, nicotinamide adenine dinucleotide (NADH), cytochrome oxidase (COX), succinate dehydrogenase (SDH), myoadenylate deaminase (MADA), aldolase and phosphofructokinase (PFK) was normal. **Immuno-histochemical studies** of skeletal muscle showed changes suggestive of a mild chronic myopathy. Immunostaining for dystrophin, dysferlin, emerin and laminin, however showed no obvious abnormality.

Mitochondrial gene sequencing was undertaken on whole blood, buccal cells and skeletal muscle biopsy from P4. She was found to be homoplasmic for several known mitochondrial polymorphisms and in addition, she was found to be homoplasmic for a novel sequence variant m.9751T>C in the *MT-CO3* gene which would result in the substitution of the normal phenylalanine residue at amino acid position 182 of the protein for a serine, predicted to be a benign substitution. The findings do not unequivocally exclude a mitochondrial disorder since mutations in nuclear genes associated with mitochondrial disorders have not been excluded.

Lamin gene sequencing was undertaken on all 12 exons of the lamin A/C gene (LMNA) and also all coding regions of the lamin-associated protein 2 gene *TMPO* (LAP2) together with flanking intronic

sequences. No mutations were detected. Both surviving children appear to have inherited the paternal LMNA allele (by SNP analysis). Of the two deceased children, one also has the paternal allele (determined by analysis of DNA extracted from FFPE tissue) and the other may also have this allele, although sequencing was inconclusive. This is relevant if the disorder is postulated to be transmitted from the mother.

Urine organic and amino acids on P4 showed no abnormality. **Blood spot acylcarnitine profiling** also showed no abnormality. **Whole blood carnitine** also on P4 was within normal limits at 23 µmol/L (normal 11-58). **Amino acid profile** was normal. It was cautioned, however that a normal acylcarnitine profile does not unequivocally exclude a fatty acid oxidation defect, particularly if a sample has been taken during a period when the she is well.

Acetaldehyde dehydrogenase ALDH2: c.1510G>A (p.Glu504Lys), the alcohol "flushing" polymorphism common in Asian populations, as a possible trigger with the observed alcohol sensitivity – not detected in father, mother or both surviving siblings.

Family 2 (P5-P7)

The family are Tamil people from Sri Lanka living in Switzerland, the parents are first cousins. All 3 children of this family were affected by the same neonatal lethal condition.

P5 (PPA2: c.[500C>T];[500C>T], p.[Pro167Leu];[Pro167Leu])

The boy was born spontaneously in 1996 after an uneventful pregnancy. Birth weight was 2820 g, length 49 cm, head circumference 33.5 cm and Apgar scores 9/10/10. The first days of life were uneventful, mother and child left the hospital on day 6. The baby was breast-fed without problems. On the 10th day of life he vomited once and loose stools were observed. On the next morning the child vomited once more but otherwise his clinical status was unremarkable. On the same afternoon (11th day of life) the child was readmitted with signs of tonic clonic seizures. The muscular hypotonic boy was somnolent and pale. Heart and lung function seemed normal initially. Lactate was elevated at 10.5 mmol/l (normal 0.4-2.8 mmol/l). In the following hours he showed again tonic-clonic seizures, which could be suspended just for a while by treatment with Diazepam and Phenobarbital. Oxygen saturation was persistently low necessitating intubation, but even under artificial respiration this did not improve and generalised tonic clonic seizures persisted. ECG investigation showed a convex ST segment elevation. Few hours later the child died with severe bradycardia.

Investigation of plasma amino acids showed elevated alanine. Investigation of organic acids was normal. Acute myocarditis was suspected.

Investigation of heart autopsy revealed areas of fresh myocardium necrosis mainly of the right heart and interstitial lymphocyte infiltration. Electron microscopy of the heart showed mitochondria with degeneration of cristae but no evidence of viral infection. Microbiological investigations of all body fluids revealed no abnormalities.

P6 (PPA2: c.[500C>T];[500C>T], p.[Pro167Leu];[Pro167Leu])

The girl was born in 1997 after normal gestation and birth with good postnatal adaptation. At the age of 14 days, 2 hours after an uneventful routine check by a paediatrician, the child suddenly deteriorated. She was admitted to the intensive care unit with marked tachypnoea after having vomited twice and having suffered from two generalized seizures. Muscle tone was slightly hypotonic and a marked metabolic acidosis with a blood pH of 6.9 (normal 7.35-7.45), HCO_3 at 4 mmol/l (21-26 mmol/l), lactate 22 mmol/l and pyruvate 253 mmol/l (normal 84-784) was found. There was slight improvement with intravenous bicarbonate and glucose treatment. Additionally a vitamin cocktail was given but subsequently there was cardio-respiratory decompensation and the girl died 6 hours after admission.

Autopsy revealed bilateral acute and subacute necrosis of the myocardium, which was more prominent in the right heart. Electron microscopy of the heart showed mitochondria with degeneration of cristae as seen in the brother (P5). Skeletal muscle was normal. Furthermore multiple subacute necroses in both cerebral hemispheres were found. Investigation of the respiratory chain enzymes and pyruvate dehydrogenase were normal in skeletal muscle and fibroblasts. Organic acids and amino acids in urine and plasma were normal. Screening of mitochondrial DNA from heart and liver did not reveal any pathological findings.

P7 (PPA2: c.[500C>T];[500C>T], p.[Pro167Leu];[Pro167Leu])

This boy was born in 2000 at term after an uncomplicated pregnancy. Birth weight was 3240 g. He was hospitalized in intensive care from the first minute of life and carefully observed. During the first two days he was well, similar to his siblings. Then he started to show progressive sweating, occasional vomiting and elevation of some metabolic parameters as lactate, transaminases, lactate dehydrogenase, creatine kinase, and creatine kinase-MB levels. Assuming that the siblings might have suffered from a defect in the respiratory chain isolated to the cardiac muscle this boy was treated with a cocktail supplement of vitamins usually given in defects of the respiratory chain (coenzyme Q_{10}, riboflavin, vitamins C, E, carnitine, biotin, beta-carotene). Over the next few days he became exhausted during feeding and developed signs of slight cardiac failure. Selective screening for inborn errors did not reveal any pathological findings. Plasma lactate remained within the normal range.

On day 9, the boy was additionally treated with thiamine hydrochloride i.v., 20 mg/d since some symptoms resembled Beri-Beri and marked improvement of his condition was noted. Thiamine was unfortunately discontinued on day 11 since he seemed to be perfectly well. However, his condition worsened again and heart failure became evident with occasional arrhythmia. Levels of troponin and transaminases increased. Echocardiography showed impaired function of the enlarged right ventricle. On day 15 it was decided to supplement him with thiamine 3 x 100 mg/day orally. Heart function improved, and the troponin and transaminase level normalized. However, on day 17 recurring tachycardia occurred, which responded temporarily to adenosine and then to electroconversion, but it recurred over the following days. A regular sinus rhythm could be obtained after treatment with boli of 5-20 mg i.v. thiamine and the boy clinically improved dramatically. However, in spite of thiamine 30-80 mg i.v. daily and propafenone, severe arrhythmia (Hf approx. 140 bpm), which turned out to be ventricular, became a serious problem while cardiac function remained stable and troponin and transaminase levels were normal. On day 30, ventricular arrhythmia persisted and did not respond to lidocaine and electroconversion. The child was neurologically normal for his age, alert and fine. He died in the early morning of the 32nd day of life from untreatable arrhythmia. A final echocardiography showed a hypodynamic right ventricle, while the left ventricle was still in a sufficient status (SF about 28-30%). A defect of thiamine metabolism/transport was considered, however, later studies with fibroblasts (by Ellis Neufeld, Boston) revealed normal thiamine uptake and conversion to thiamine pyrophosphate.

Autopsy revealed a myocardium without necrosis and inflammatory infiltrations. Myocytes with reduced amount of myofibrils were found. In the myocardium of the right heart there was a herd of fibrosis. Investigation of the respiratory chain in autopsy samples of the heart showed a moderate decrease of complex I 4.1 mU/mg protein (normal 5.5-51.5 mU/mg protein) and complex IV 64 mU/mg protein (normal 73.2-516.6) in the left ventricle. In the right ventricle the activity of complex I was not detectable, complex II, 9.0 mU/mg protein (normal 73.2-516.6), and complex IV, 42 mU/mg protein were reduced. Normal activities were found in skeletal muscle and fibroblasts. Investigation of the mitochondrial DNA did not reveal pathogenic mutations.

Family 3 (P8-P9)

This is a multiply consanguineous family of Pakistani origin, living in the UK. The parents are first cousins.

P8 (PPA2: c.[500C>T];[500C>T], p.[Pro167Leu];[Pro167Leu])

Their first child (II:1) was seemingly well with normal growth and development until the age of 5 ½ months. She was then admitted to hospital following a 24 hour history of vomiting and diarrhoea and

had suffered a seizure at home. She had further seizures on arrival at A&E and a poor response to treatment, so was intubated and ventilated. A CT head scan was normal. Cardiac echocardiogram showed poor contractility and a small amount of tricuspid regurgitation. She was transferred to Sheffield Children's Hospital and had a cardiac arrest on route. She then suffered further multiple cardiac arrests and despite maximal attempts at resuscitation she eventually died during the course of these.

On post mortem examination there were no specific macroscopic abnormalities. Infection screen identified rotavirus in the stool. The brain showed hypoxic injury. The liver showed mild fatty change. Skeletal survey, metabolic and toxicology screens were normal. Fatty acid oxidation levels were carried out on skin fibroblasts and were normal. The heart appeared normal in size, shape and structure. Histology of the heart showed areas of recent necrosis, thought to be related to the recent cardiac arrests. There was also evidence of long-standing myocyte loss with increased interstitial collagen and focal myocyte fibre disarray in the left ventricle and interventricular septum. The disarray was considered insufficient for a diagnosis of Hypertrophic Cardiomyopathy. Tests for myocarditis were normal. CSF glucose was low, but this was performed on a post mortem sample.

Their second child (II:2) is fit and well and is now 4 years of age. A recent echocardiogram was normal, as were lactate, acylcarnitine and CK.

P9 (PPA2: c.[500C>T];[500C>T], p.[Pro167Leu];[Pro167Leu])

Their third child (II:3) suffered a viral illness at the age of 8 months and then suffered a week's history of increasing hypotonia and weakness. CK was 15,000 at this time and plasma lactate was raised at 5. Free carnitine was normal at 43.6, but propionylcarnitine raised at 2.38 (<1.5). Urine organic acids were normal. There was no involvement of respiratory muscles. Renal function was normal. Over a period of two weeks her weakness and hypotonia improved and her CK reduced. Echocardiogram at this time was normal.

She presented again at the age of 11 months to hospital with diarrhoea and vomiting, her oral intake was poor and she was not passing urine. She became increasingly drowsy and capillary refill was prolonged at 3 seconds. An initial blood gas showed pH 6.9, bicarbonate 13.8, base excess -13.1, lactate 8.7 and glucose 6.18. She then suffered a focal seizure with lateral gaze to the left and left sided upper limb jerks, which subsequently generalized and lasted for 12 minutes. She was given IV Lorazepam, a fluid bolus, IV antibiotics and acyclovir. Further seizures followed which were treated with IV Lorazepam, Phenytoin and PR Paraldehyde. At three hours following admission seizures had settled but she was still drowsy. CT head scan at this time was normal. Not long after this she

suffered a further focal seizure involving the left upper limb. She then suffered a cardiac arrest and was intubated and ventilated. Maximal CPR was continued for 20 minutes but was not successful.

Post mortem examination showed very extensive fibrosis of the heart muscle and normal appearance of the skeletal muscle. The brain looked normal at post mortem. Norovirus infection was confirmed on stool samples from admission.

Respiratory chain analysis on peripheral muscle tissue was normal, and histological and histochemical assessment of muscle biopsy did not reveal any major mitochondrial abnormalities.

Family 4 (P10)

This family lives in Northern Ireland, the parents are non-consanguineous. The first of their two children was affected by fatal childhood disease.

P10 (PPA2: c.[380G>T];[514G>A], p.[Arg127Leu];[Glu172Lys])

Individual P10 is a male and was the first child born to non-consanguineous parents. He was born by emergency caesarean section for failure to progress but was not admitted to the special care baby unit. His birth weight was 4.59 kg. He had some feeding difficulties in his first year and was on Nutramigen for possible allergies. His feeding settled and he was changed onto normal milk at 6 months of age. He had an admission at 10 months with a short seizure. This settled spontaneously and he was observed overnight.

He had a prolonged admission to PICU at 1 year of age following a coryzal illness when he developed cardiomyopathy, multiorgan failure and rhabdomyolysis requiring inotropic support and dialysis. He was intubated and ventilated for over 3 weeks. Initial metabolic investigations suggested an underlying VLCAD deficiency (his acylcarnitine profile was abnormal with elevated C14:1, C14 and C16:1 suggestive of VLCAD, MIM: 609575), but excluded on fatty acid oxidation studies from Sheffield. CT brain was normal. ECHO showed markedly dilated left ventricle with moderate decrease in left ventricular function. Viral myocarditis was considered, but no virus was isolated. His urinary organic acids showed large increase in 3 hydroxybutyrate and acetoacetate. Plasma amino acids were essentially normal.

Exome sequencing revealed a heterozygous mutation in *ACADVL* (NM_000018.3): c.1844G>A, p.(Arg615Gln), which is a variant of unclear clinical relevance. In the ExAC consortium this mutation is found in 345 of 121088 alleles (allele frequency 0.002849) in heterozygous state and two individuals are homozygous in this collective. Minimal coverage of *ACADVL* was 11-fold in exome analysis.

On transfer to the wards they had concerns about his neurocognitive state. MRI brain showed no structural abnormality but mild enlargement of the ventricular system in keeping with an atrophic process. MR spectroscopy was normal. He had a normal eye examination. Brain stem auditory evoked responses were normal. He had intensive physiotherapy and made a good recovery, but had some central weakness. He was discharged from hospital at 14.5 months (admitted for 2.5 months). Repeat ECHO prior to discharge showed normal left ventricular function with a degree of muscle thickening.

His health was reasonably good and he attended the Child developmental clinic and he appeared to show some regression in terms of communication and social interaction, and had bilateral alternating squint. Paediatric cardiology review and echocardiogram at 18 months showed good systolic function with an ejection fraction of 74% and mild left ventricular hypertrophy.

At 2 years of age, he was admitted with vomiting and diarrhoea secondary to Norovirus infection. He deteriorated over the course of a day and developed a mixed metabolic and respiratory acidosis and afebrile seizures. He was transferred to PICU and unfortunately had an asystolic cardiorespiratory arrest with no response to resuscitation.

Respiratory chain analysis on peripheral skeletal muscle showed no evidence of a mitochondrial respiratory chain defect; muscle biopsy showed no evidence of myopathic or neurogenic disorder, no fibre variation, necrosis, inclusions, ragged-red or cytochrome c oxidase-deficient fibres.

A metabolic post-mortem was performed (Figure S1). Cause of death was due to cardiac failure secondary to myocardial fibrosis and acute myocardial ischaemia due to mitochondrial myopathy. The post-mortem heart was enlarged (86 g in weight) with evidence of mild hypertrophy of the left ventricular wall which was 1.0 cm in thickness. There was transmural irregular pallor and fibrosis on sectioning and the myocardium was stiff and dense in texture although no evidence of endocardial fibroelastosis on naked eye examination. Routine histology revealed bilateral pleural effusions and a pericardial effusion with pulmonary oedema. There was extensive transmural fibrosis of the left ventricle and the septum of the heart with board swathes of fibrotic tissues replacing the myocytes. Considerable myocyte nuclear pleomorphism and hyperchromasia with variation in myocyte size was noted, consistent with transmural fibrosis secondary to ischaemic injury. Endocarditis was not observed, whilst the coronary arteries showed no vasculitis or thrombosis. As documented in the main manuscript text, assessment of respiratory chain enzyme activities in a cardiac muscle sample

revealed a significant and isolated mitochondrial respiratory chain defect involving complex I in isolation.

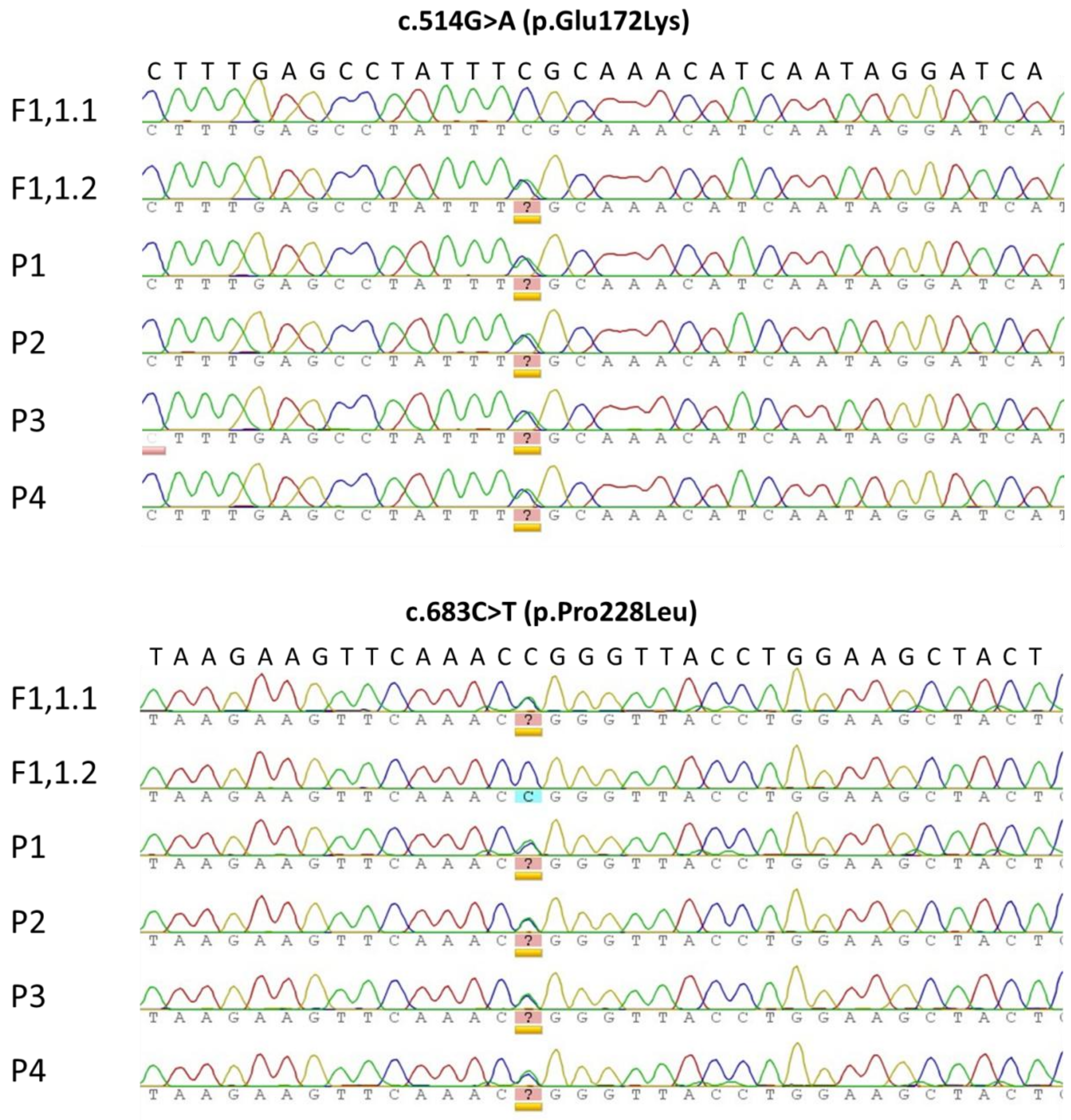

Figure S1. Sanger sequencing in Family 1 revealed compound heterozygous mutations in *PPA2* in all affected individuals.

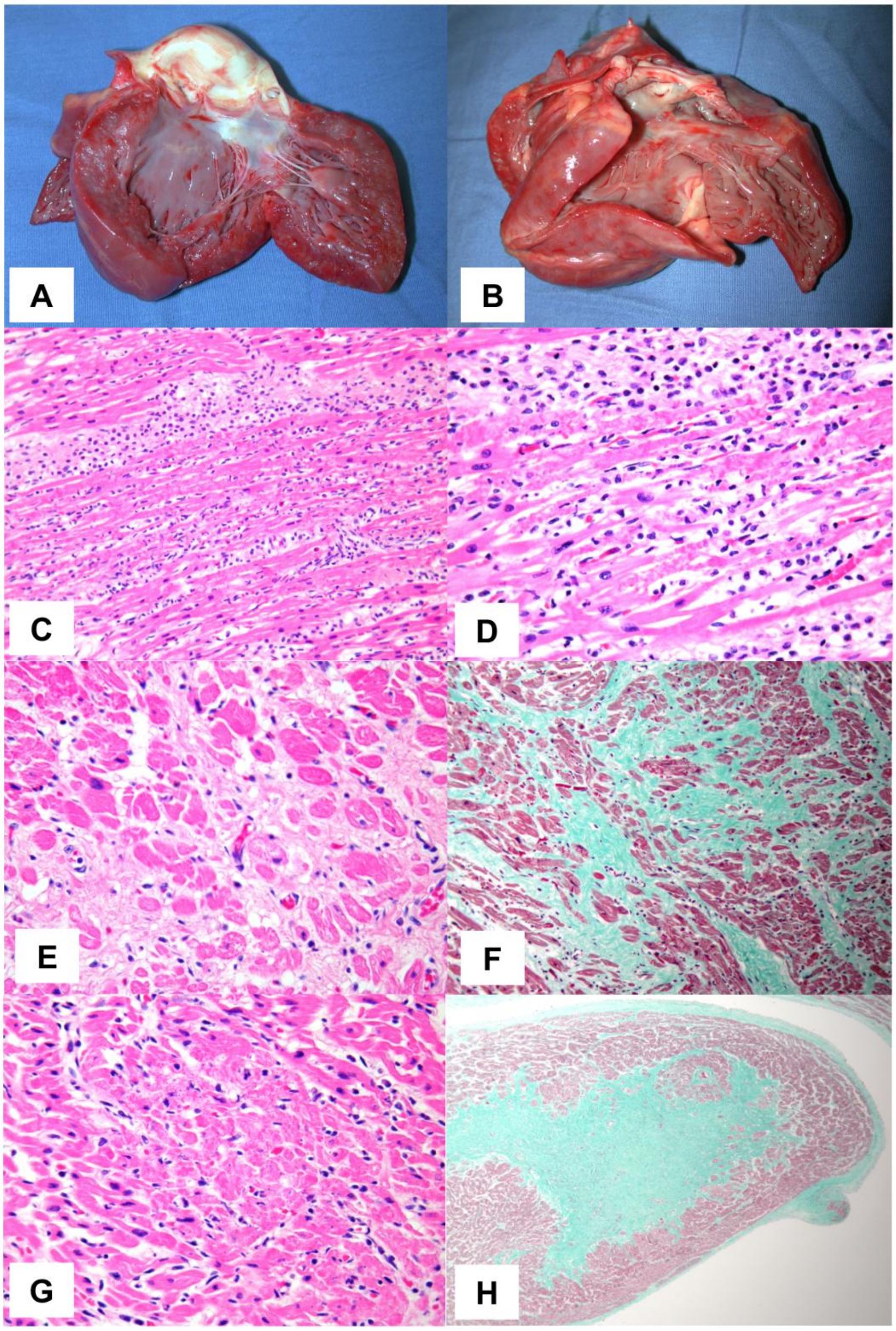

Figure S2. Post-mortem findings in the cardiac muscle from Patient 10. (A, B) Gross examination revealed an enlarged heart with evidence of left ventricular hypertrophy. The myocardium was stiff and firm, with extensive areas of pallor noted in this tissue. (C) Low and (D, E) higher power haematoxylin and eosin (H&E) staining revealed areas of acute inflammatory infiltrate between and around cardiac muscle cells, with evidence of acute myocyte necrosis (panels C and D) as well as evidence of older degenerative changes including fibrosis and nuclear pleomorphism (E). Masson trichrome staining confirms extensive fibrosis, with areas of cardiac muscle (staining red-purple color) replaced by collagenous fibrotic tissue (green-teal color) (F). Additional pathological findings include areas of early ischaemic necrosis that can be easily differentiated from the less-visibly damaged cardiac muscle cells around it (G) and severe fibrosis of a papillary muscle (Masson trichrome stain) which might be implicated in the observed valvular dysfunction (H).

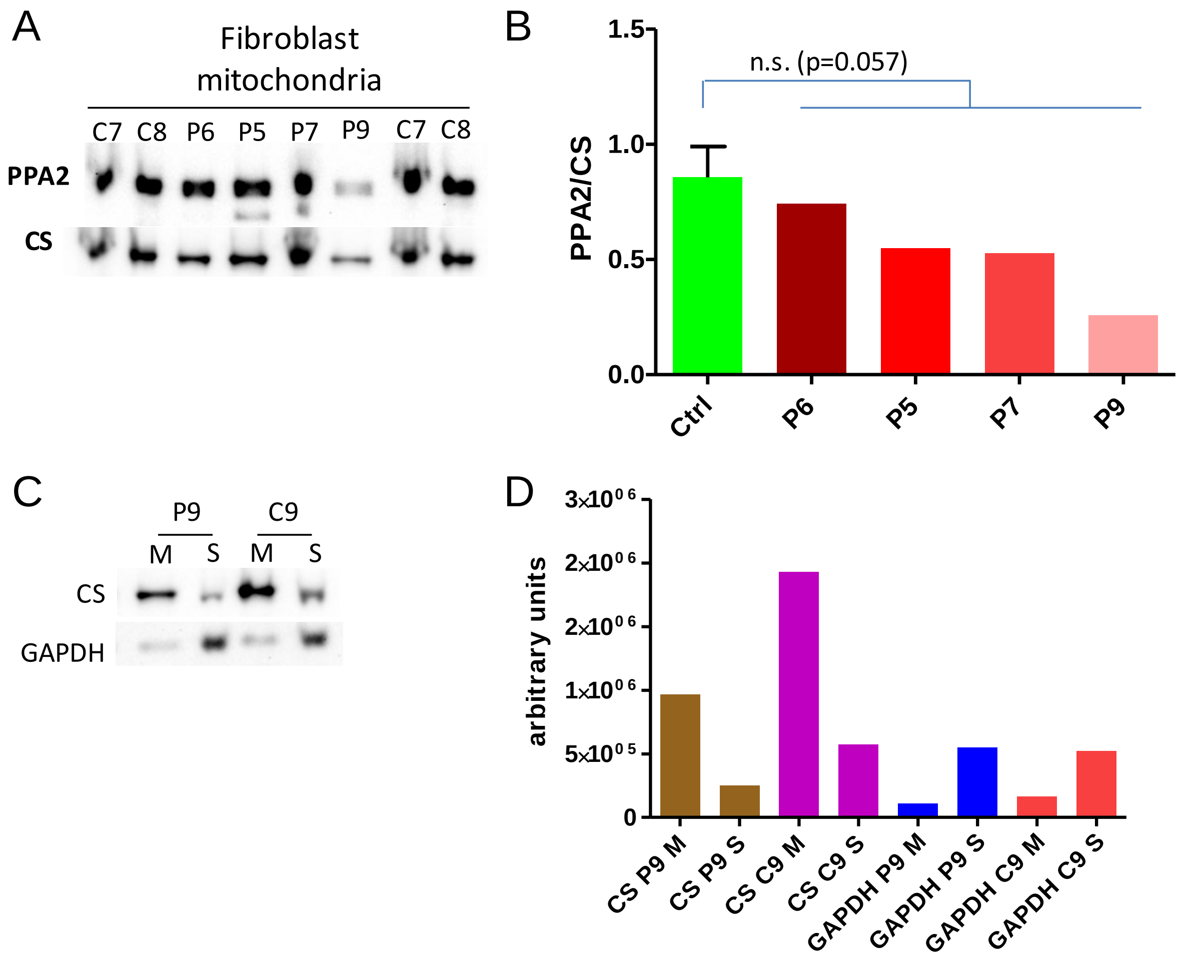

Figure S3. Western blot analysis from mitochondria isolated from fibroblasts. Isolated mitochondria form fibroblasts were available from affected individuals P5, P6, P7 and P9. Antibodies against PPA2 and citrate synthase (CS) were used (A-B). Antibodies against citrate synthase and glyceraldehyde 3-phosphate dehydrogenase (GAPDH), cytosolic housekeeping protein, were used (C-D). The supernatant (S) of mitochondria (M) isolation was investigated in individual P9, which showed only small amounts of cross contaminations of mitochondria with cytosolic protein (C-D). Statistical analysis was performed by Student's unpaired t-test. The error bar indicates the standard error of the mean (B). Abbreviation: n.s., not significant.

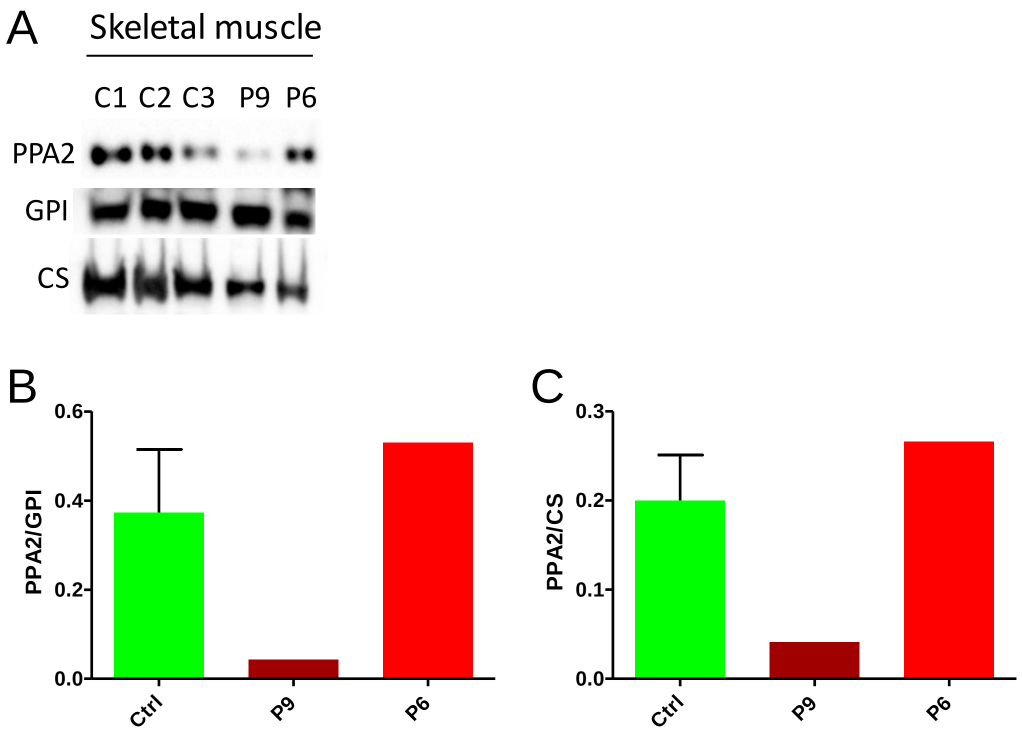

Figure S4. Western blot analysis from 600 g supernatants of skeletal muscle. Frozen skeletal muscle autopsy samples were available from affected individuals P6 and from P9. Antibodies against PPA2, citrate synthase (CS), mitochondrial matrix protein, and glucose-6-phosphate isomerise (GPI), cytosolic housekeeping protein, were used (A). Relative ratios of PPA2/GPI (B) and PPA2/CS (C) were quantified. The error bars in this graph indicate the standard error of the mean.

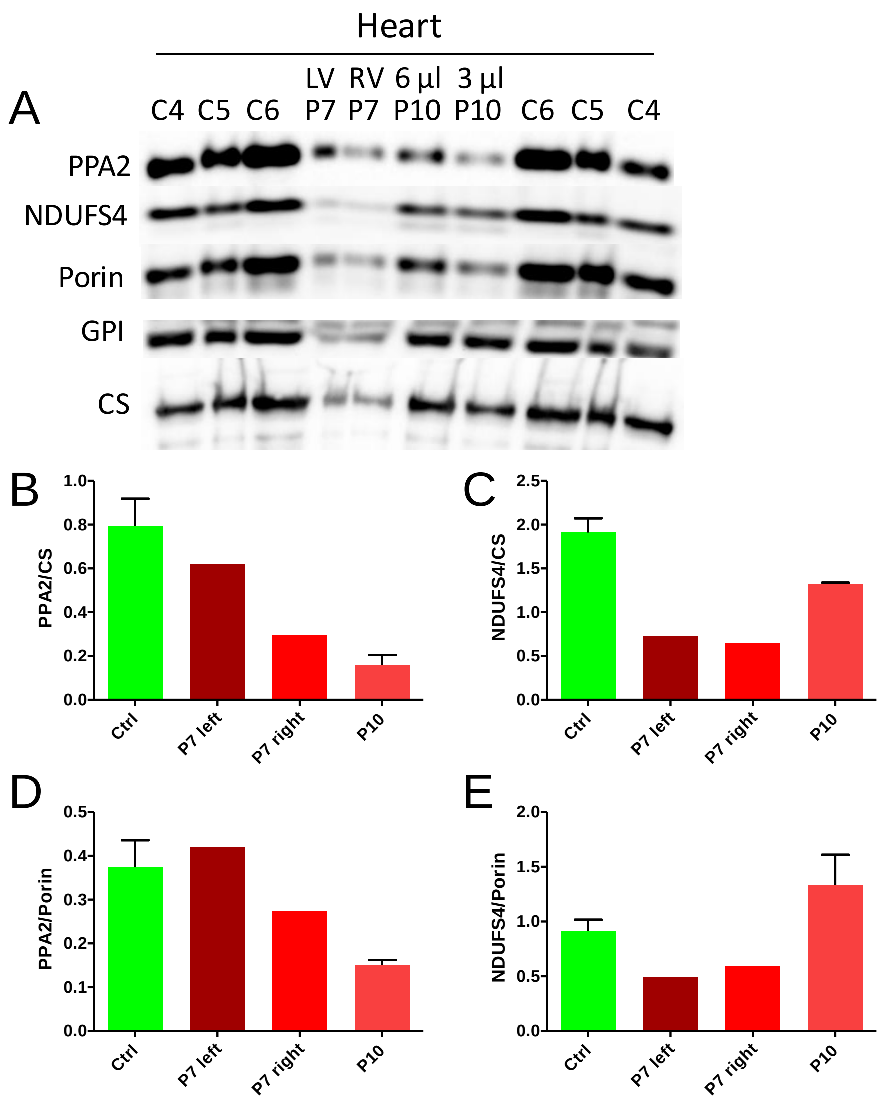

Figure S5. Western blot analysis from 600 g supernatants of heart homogenates. Frozen heart autopsy samples were available from affected individuals P7, left (LV) and right ventricle (RV), and from P10. Antibodies against PPA2, NDUFS4, subunit of complex I, citrate synthase (CS), mitochondrial matrix protein, porin, mitochondrial outer membrane protein, glucose-6-phosphate isomerise (GPI), cytosolic housekeeping protein, were used (A). Relative ratios of PPA2/CS (B), NDUFS4/CS (C), PPA2/porin (D), and NDUFS4/porin (E) were quantified. The error bars in this graph indicate the standard error of the mean.

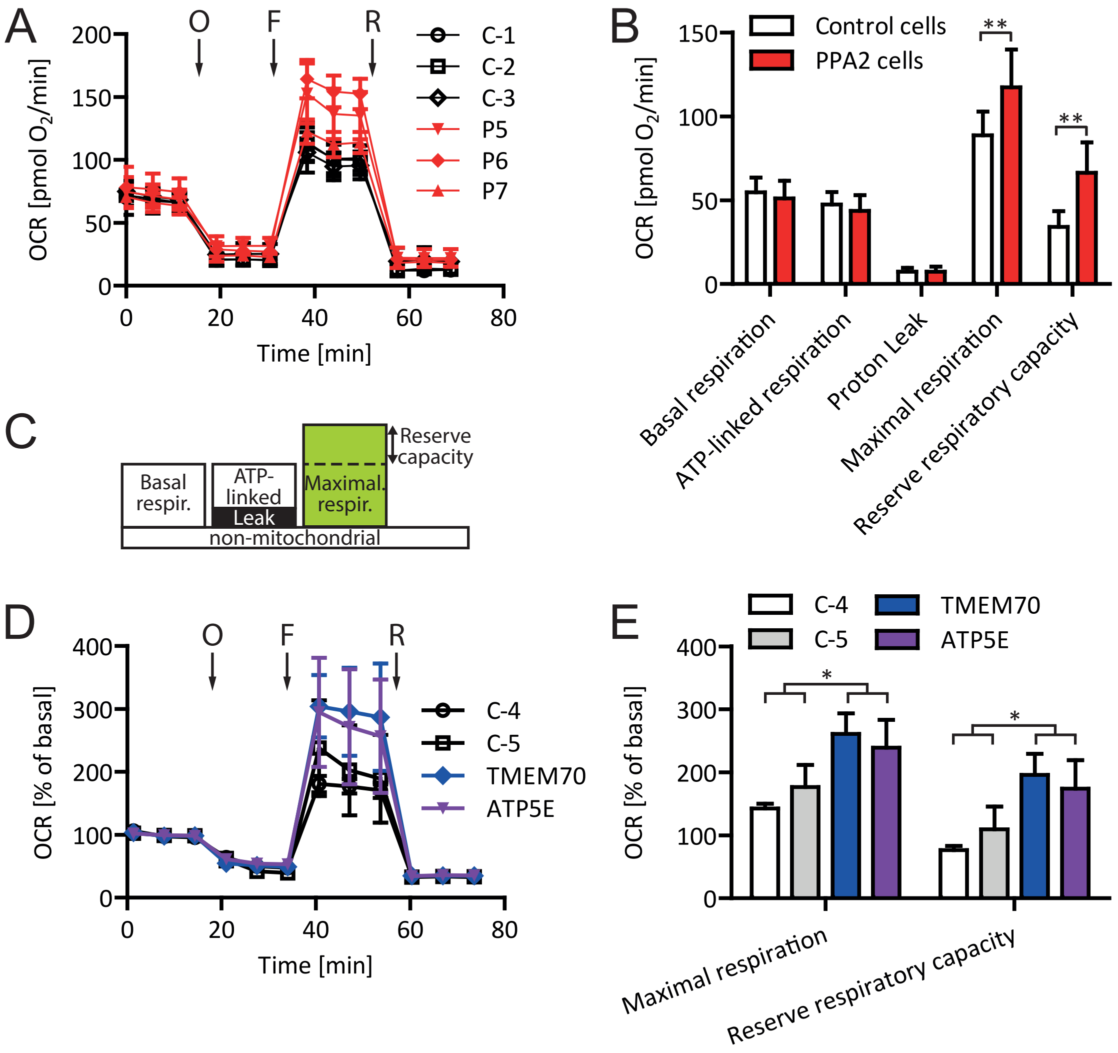

Figure S6. Oxygen consumption rates of PPA2 deficient fibroblasts. (A) Oxygen consumption rates (OCR) have been determined in three control (C-1 to C-3) and three affected with PPA2 mutations (P5, P6, and P7) and revealed increased maximal respiration as well as reserve respiratory capacity (B, C). Fibroblasts from individuals with proven ATP synthase deficiency and mutations in either TMEM70 and ATP5E showed a similar result with increased reserve respiratory capacity (D, E). Oligomycin (O, 1.0 µmol/l), carbonyl cyanide-4-(trifluoromethoxy)-phenylhydrazone (F, 0.4 µmol/l) and rotenone (R, 2.0 µmol/l) were added during the experiment. *$P<0.01$, **$P<0.0001$ in Student's unpaired t-test. The error bars in this graph indicate the standard error of the mean.

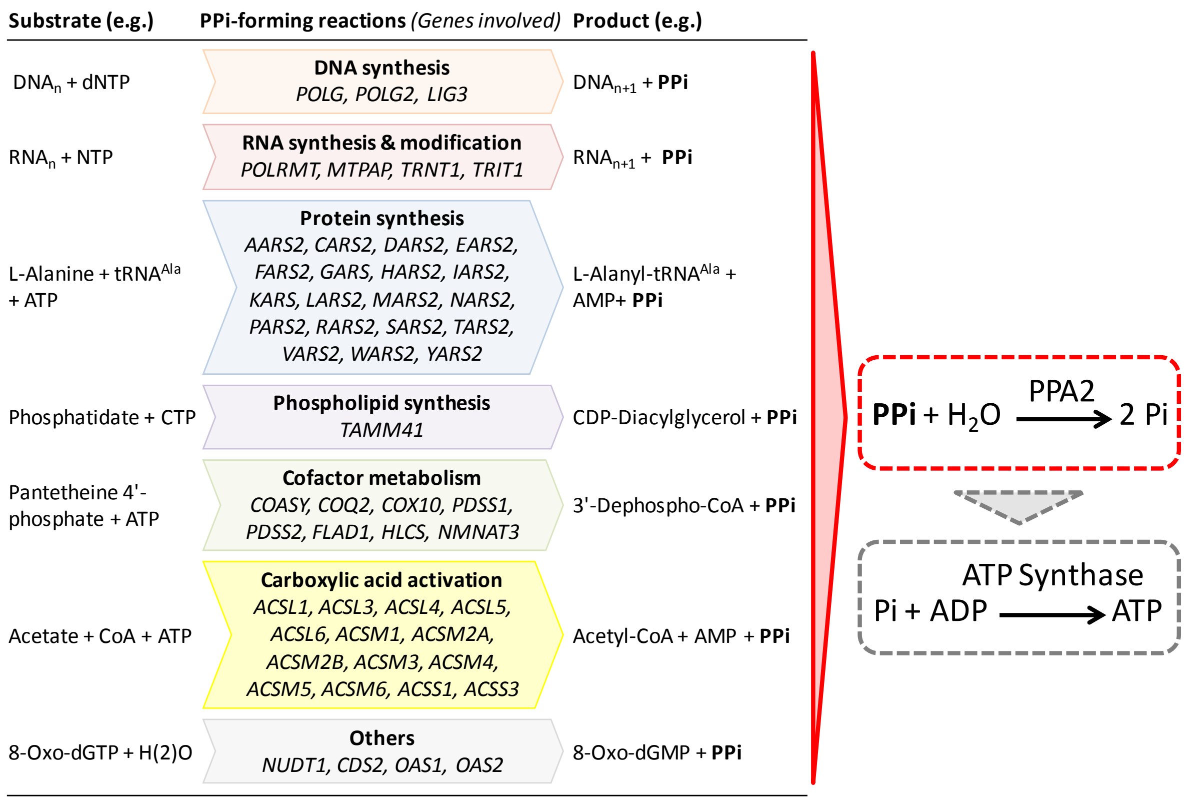

Figure S7. Reactions in Mitochondria Upstream and Downstream of Inorganic Pyrophosphate (PPi). Abbreviations: Deoxynucleotide triphosphate (dNTP), deoxynucleotide monophosphate (dAMP), nucleotide triphosphate (NTP), nucleotide monophosphate (NMP), adenosine triphosphate (ATP), adenosine diphosphate (ADP), adenosine monophosphate (AMP), cytidine triphosphate (CTP), cytidine monophosphate (CMP), Coenzyme A (CoA), deoxyguanosine triphosphate (dGTP), deoxyguanosine monophosphate (dGMP), orthophosphate (Pi), mitochondrial pyrophosphatase (PPA2).

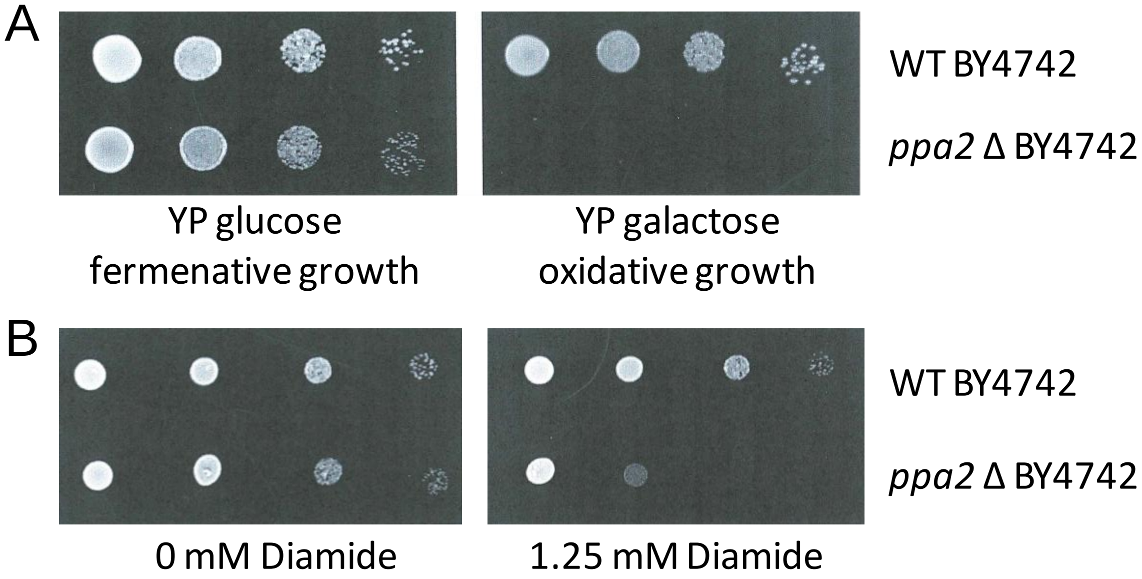

Figure S8. Investigation of yeast PPA2 knock-out cells. Growth defect of a wild type (WT BY4742) and a PPA2 knock-out (*ppa2* Δ BY4742) *S. cerevisiae* strain on aerobic medium showing that PPA2 is critical for mitochondrial respiration (A). Oxidative stress sensitivity of PPA2 knock-out *S. cerevisiae* as measured by growth defect of diamide, which oxidises intracellular thiols and mimics oxidative stress in yeast (B).

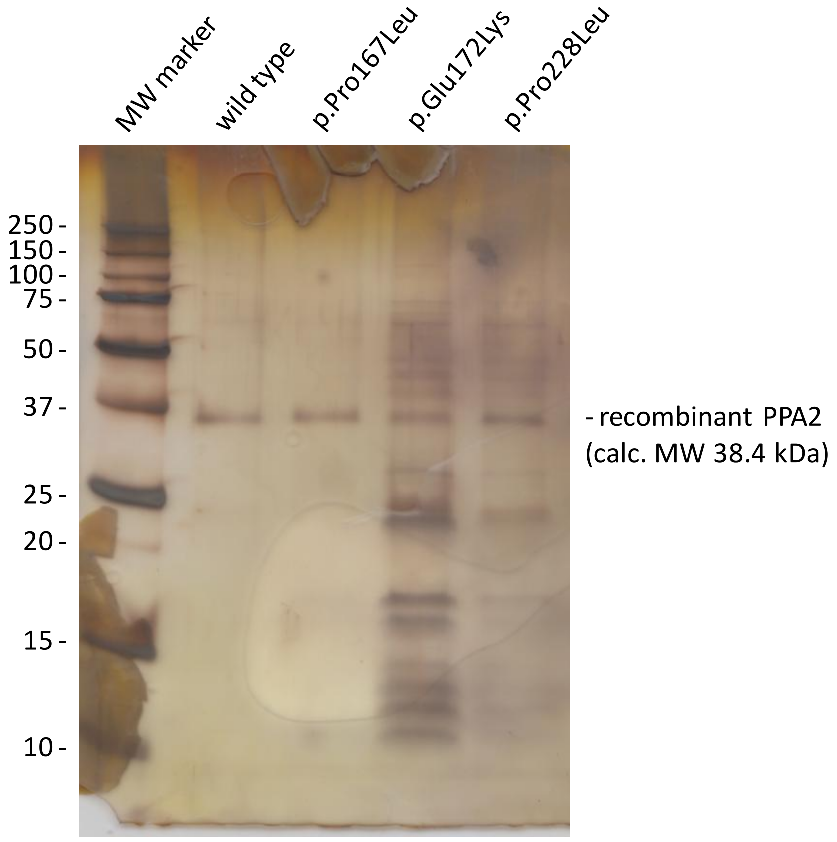

Figure S9. Silver staining of recombinant His-tagged purified PPA2 protein. The same volumes of recombinant protein as used for western blotting in Figure 3G were loaded on a 10% SDS polyacrylamide gel.

Table S1. Information on exome sequencing performed in three different centers.

Sequencing Centre:	Christchurch, NZ								
**Individual ID**	**Instrument**	**Library Prep**	**Exome Capture**	**Reads**	**Mapped**	**% Mapped**	**Mean cov**	**Cov 30x**	**Variant annotation method**
F1, 1.1	Illumina HiSeq® 2000	Illumina TruSeq® DNA LT	Illumina TruSeq® Exome Enrichment	158461290	51814344	87,15	50.15	95,15	Illumina HiSeq® analysis software enrichment pipeline v.0.9, followed by ANNOVAR and ENCODE Gencode v.12 (GRCh37). Variants were sequentially filtered against the 1000 Genomes (Apr 2012 release) and dbSNP137 databases. Variants with a minor allele frequency >0.01 were removed.
F1, 1.2	Illumina HiSeq® 2000	Illumina TruSeq® DNA LT	Illumina TruSeq® Exome Enrichment	125910016	42782905	86,58	39.75	95,33	
P2	Illumina HiSeq® 2000	Illumina TruSeq® DNA LT	Illumina TruSeq® Exome Enrichment	177926998	63271018	87,43	59,27	95,11	
P4	Illumina HiSeq® 2000	Illumina TruSeq® DNA LT	Illumina TruSeq® Exome Enrichment	147463447	43937929	87,95	42,45	94,95	

Sequencing Centre:	Munich, GER								
**Individual ID**	**Instrument**	**Library Prep**	**Exome Capture**	**Reads**	**Mapped**	**% Mapped**	**Avg cov**	**Cov 20x**	**Variant annotation method**
P6, 85154	Illumina HiSeq® 2500	SureSelect XT Target Enrichment system for Illumina	SureSelectAllExon v5	113371747	112668276	99.38	144.71	97.40	Reads were aligned to genome assembly hg19 with Burrows-Wheeler Aligner (BWA, V.0.5.87.5) and genetic variation was detected using SAMtools (V.0.1.18), PINDEL (V.0.2.4t) and ExomeDepth (V.1.0.0). Candidate genes were prioritized by searching for homozygus or potentially compound heterozygous variants with a minor allele frequency < 1% in 7,000 in-house control exomes, dbSNP, 1000 Genomes and ExAC.
P5, 85155	Illumina HiSeq® 2500	SureSelect XT Target Enrichment system for Illumina	SureSelectAllExon v5	120884660	120092778	99.34	151.17	97.74	
P7, 85152	Illumina HiSeq® 2500	SureSelect XT Target Enrichment system for Illumina	SureSelectAllExon v5	121393141	120644253	99.38	152.67	97.79	
P10, 68551	Illumina HiSeq® 2500	SureSelect XT Target Enrichment system for Illumina	SureSelectAllExon v5	103709389	102998478	99.31	129.11	97.19	

Sequencing Centre:	Leeds, UK								
**Individual ID**	**Instrument**	**Library Prep**	**Exome Capture**	**Reads**	**Mapped**	**% Mapped**	**Mean cov**	**Cov 20x**	**Variant annotation method**
P8 (JT609)	Illumina HiSeq® 2500	SureSelect XT Target Enrichment system for Illumina	SureSelectAllExon v5	72844614	71182311	97.71	73.41	90.8	In house pipeline: Alignment carried out using Novoalign. Variants were called using the HaplotypeCaller (GATK, Broad Institute) .vcf files were annotated using Ensembl's Variant Effect Predictor (VEP). Local Perl scrips were used to remove variants present at >1% minor allele frequency in the following databases: dbSNP 138 and previous, NHLBI Exome Sequencing Project
P9 (JT579)	Illumina HiSeq® 2500	SureSelect XT Target Enrichment system for Illumina	SureSelectAllExon v5	74044360	72332089	97.68	72.14	90.6	
I:1 (JT576)	Illumina HiSeq® 2500	SureSelect XT Target Enrichment system for Illumina	SureSelectAllExon v5	73769160	72104134	97.74	76.35	90.2	

I:2 (JT577)	Illumina HiSeq® 2500	SureSelect XT Target Enrichment system for Illumina	SureSelectAllExon v5	66440752	64892409	97.67	62.66	88.7	(ESP) Exome Variant Server, the Exome Aggregation Consortium (ExAC), and over 3000 ethnically-matched control samples. Variants were retained if predicted 'pathogenic' by any one of Polyphen2, SIFT or condel. Variants were ordered by CADD score and those with CADD score >15 were retained.
II:2 (JT578)	Illumina HiSeq® 2500	SureSelect XT Target Enrichment system for Illumina	SureSelectAllExon v5	59183468	57848931	97.75	62.23	87.7	

Table S2. Compounding missense mutations in cardiomyopathy/mitochondrial associated genes, excluded from further analysis due to non-segregation within Family 1.

GENE	VARIANT			EXAC FREQUENCY	SEGREGATION WITH PHENOTYPE?
KCNJ12	rs1657740	NM_021012.4(KCNJ12):c.353G>A	p.Arg118Gln	0.4994 (60403/120958)	NO
	rs77048459	NM_021012.4(KCNJ12):c.715G>A	p.Glu239Lys	0.2807 (27048/96360)	NO
TTN	rs56341835	NM_001267550.1(TTN):c.10213G>A	p.Glu3405Lys	0.0007518 (90/119710)	NO
	rs142094090	NM_001267550.1(TTN):c.50515G>A	p.Glu16839Lys	0.0007932 (96/121024)	NO
AARS2	rs79962181	NM_020745.3(AARS2):c.1649G>C	p.Gly550Ala	0.0007925 (96/121138)	NO
	rs142094090	NM_020745.3(AARS2):c.1621G>A	p.Glu541Lys	0.0007932 (96/121024)	NO

Table S3. Prediction Tools - Estimation of pathogenic relevance

1. Prediction for PPA2 mutations identified by exome sequencing

**Mutation**<br>**Protein**	**c.380G>T**<br>**p.Arg127Leu**	**c.500C>T**<br>**p.Pro167Leu**	**c.514G>A**<br>**p.Glu172Lys**	**c.683C>T**<br>**p.Pro228Leu**
Family	F4	F2, F3	F1	F1, F4
ExAC, heterozygotes (total alleles)	20 (121354)	3 (120914)	59 (120800)	30 (120268)
SIFT Prediction	DAMAGING	DAMAGING	DAMAGING	DAMAGING
SIFT Score (deleterous when <0.05)	0.00	0.01	0.00	0.00
PolyPhen-2 Prediction	PROBABLY DAMAGING	PROBABLY DAMAGING	PROBABLY DAMAGING	PROBABLY DAMAGING
PolyPhen-2 (score)	0.993 (sensitivity: 0.70; specificity: 0.97)	1.000 (sensitivity: 0.00; specificity: 1.00)	0.996 (sensitivity: 0.55; specificity: 0.98)	1.000 (sensitivity: 0.00; specificity: 1.00)
Mutation Taster Prediction	disease causing	disease causing	disease causing	disease causing
Mutation Taster (probability)	0.99999999999608	0.999999999999996	0.99999999999269	0.999999999999993
Mutation Taster (predicted change)	1-amino acid sequence changed, 2-protein features (might be) affected, 3-splice site changes	1-amino acid sequence changed	1-amino acid sequence changed	1-amino acid sequence changed

2. Results for homozygous missense mutation in PPA2 from the ExAC database

**Mutation**<br>**Protein**	**c.251G>A**<br>**p.Arg84Gln**	**c.727G>T**<br>**p.Val243Leu**	**c.846G>C**<br>**p.Lys282Asn**
ExAC, homozygotes (total alleles)	1 (116414)	6 (120536)	13349 (121124)
ExAC, heterozygotes (total alleles)	34 (116414)	280 (120536)	55039 (121124)
SIFT Prediction	TOLERATED	TOLERATED	TOLERATED
SIFT Score (deleterous when <0.05)	0.18	0.15	0.14
PolyPhen-2 Prediction	benign	benign	benign
PolyPhen-2 (score)	0.005 (sensitivity: 0.97; specificity: 0.74)	0.170 (sensitivity: 0.92; specificity: 0.87)	0.038 (sensitivity: 0.94; specificity: 0.82)
Mutation Taster Prediction	polymorphism	disease causing	polymorphism
Mutation Taster (probability)	0.997195759241821	0.999989907773341	0.999999999971653
Mutation Taster (predicted change)	1-amino acid sequence changed, 2-protein features (might be) affected, 3-splice site changes	1-amino acid sequence changed, 2-protein features (might be) affected, 3-splice site changes	1-amino acid sequence changed, 2-protein features (might be) affected, 3-splice site changes

Human PPA2 sequence IDs: GenBank transcript NM_176869.2; GenBank Protein NP_789845.1; UniProt Q9H2U2; Ensembl transcript ENST00000341695; Ensembl protein ENSP00000343885
